# Metamorphic heritage values: Revisiting architectural heritage assessment in clustered and residual contexts

**DOI:** 10.12688/openreseurope.23051.1

**Published:** 2026-02-16

**Authors:** Raffaella De Marco, Izabella Parowicz

**Affiliations:** 1 DICAr - Dept. of Civil Engineering and Architecture, University of Pavia, Pavia, Lombardia, 27100, Italy; 2 Chair for Heritage Studies, European University Viadrina, Frankfurt an der Oder, Brandenburg, 15230, Germany

**Keywords:** values assessment; architectural heritage; value typologies; heritage values; metamorphic values; clustered heritage; residual heritage

## Abstract

**Background:**

Values-based approaches are central to architectural heritage conservation, yet their institutionalisation has often transformed analytical value typologies into normative-operational templates. This stabilisation risks obscuring the dynamic, negotiated, and context-dependent nature of heritage significance, particularly in settings shaped by rupture, displacement, and coexistence. Such limitations are especially evident in residual and clustered heritage contexts, where architectural heritage persists amid discontinuity, layered memories, and competing narratives. This study addresses the need for a more flexible conceptualisation of value attribution capable of accounting for transformation, plurality, and perspective-dependence.

**Methods:**

The research adopts a qualitative, conceptual methodology combining an interdisciplinary narrative literature review with an attribute-based analysis of heritage value typologies. A genealogical mapping of value frameworks across architecture, archaeology, sociology, and economics was conducted, followed by the development of a Value–Attribute (V>A) matrix to identify shared and overlapping conceptual properties. These analytical steps were situated within a critical discourse on residual and clustered heritage contexts. Building on this foundation, the study develops a metamorphic interpretive framework to analyse how heritage values evolve, overlap, and diverge over time and across stakeholders.

**Results:**

The analysis demonstrates that many established value typologies rely on porous and overlapping definitions, which become problematic when applied as fixed assessment categories in complex socio-political contexts. The study identifies three recurrent modes of value transformation: accretive values, where meanings accumulate over time; palimpsestic values, where earlier interpretations are partially overwritten yet remain traceable; and refracted values, where meanings diverge across cultural, political, or emotional perspectives. These modes reveal how architectural heritage significance is continuously reconfigured rather than replaced, particularly in contexts marked by displacement, forced coexistence, or contested memory.

**Conclusions:**

The paper proposes metamorphic heritage values as a heuristic and reflective lens rather than a new classificatory system. By shifting attention from enumerating values to tracing their transformation, the framework supports a more adaptive, pluralistic, and context-sensitive approach to heritage assessment. This perspective enhances the capacity of values-based methodologies to engage with residual and clustered heritage and contributes to broader debates on the epistemology of heritage valuation and conservation practice.

## Introduction

Values assessment is a widely established approach in heritage conservation, formalised through conservation theories and scholarly frameworks developed over the past century. It has enabled the identification of a range of value typologies ( Avrami et al. 2000; Burgos Vargas & Mora Alonso-Muñoyerro 2022; Fredheim & Khalaf 2016), which now serve as reference models for assessing heritage significance ( Avrami et al. 2000; Throsby 2001; Cleere 1989). However, while these typologies circulate between scholarship and institutions, while simultaneously shaping conservation thinking ( Mason 2002; Jokilehto 1999; Smith 2006), they risk shifting from analytical descriptors into normative-operational checklists, thereby obscuring the dynamic, contested, and context-dependent character of heritage meaning. At the same time, many established value frameworks are intentionally open-textured and can support interpretive breadth rather than rigidity ( Olukoya 2021). Thus, it is increasingly evident that the continued application of values must account for the transformative nature of heritage and the evolving dynamics of value signification in increasingly fluid and contested contexts ( Macdonald 2013). They must also remain open to ongoing scholarly debate and reframing opportunities ( Hodder & Hutson 1986; Duval et al. 2019; Olukoya 2021; Bos et al. 2025).

This ongoing critical reflection reveals the limits of fixed value taxonomies and opens a space for rethinking how meanings in heritage evolve, overlap, and conflict. The present paper contributes to this debate by proposing a conceptual reframing through the notion of metamorphic heritage values. Rather than adding another typology, this approach describes values as transformative relations, emphasising the dynamic and negotiated nature of meaning-making in heritage. By articulating this framework, the paper positions itself as a conceptual contribution to the continuing international discourse on the politics and epistemology of valuation.

Affirming the legitimacy of values assessment, therefore, requires recognising that value assignment is a dynamic, continuously evolving process, fundamentally opposed to the consolidation of rigid, static frameworks. This variability is a necessary response to unpredictable scenarios and emerging heritage crises, where instability in governance, social composition, coexistence, or environmental conditions fosters continual shifts in the significance attributed to heritage ( Feilden 2003; Stovel 1998; Fairclough & Møller 2008).

### From analytical typologies to normative-operational checklists

The consolidation of values assessment has been reinforced by international recognition of shared human values, as reflected in foundational charters such as the Venice Charter ( ICOMOS 1964), the Nara Document on Authenticity ( ICOMOS 1994), and the Burra Charter (ICOMOS Australia 2013). In sociological terms, this is a familiar trajectory of institutionalisation: categories formulated as analytical tools become objectivated and internalised as seemingly “given” features of professional reality ( Berger & Luckmann 1966). As a result, values-based approaches are now employed both as preliminary analytical tools ( Avrami et al. 2000) and as frameworks embedded within the design processes for conservation, preservation, and intervention ( Ost & van Droogenbroeck 1998; Mason 2002). Through institutionalisation, value typologies have become integral to how stakeholders, especially in community-based approaches, interpret heritage meaning, and the assignment of a site’s significance to specific values is now often considered a decisive factor for conservation decision-making. Accordingly, the inclusion or omission of certain values in assessment processes has direct implications for defining heritage significance ( Fredheim & Khalaf 2016) and shaping a site's future ( Avrami et al. 2000). As these frameworks migrate into policy routines, they are often treated as operational templates that narrow what can be articulated, compared, or debated in valuation processes.

### Transition and transformation as a challenge to standardised values-based models

Despite their widespread use, values-based models have drawn criticism for their tendency toward rigidity and standardisation ( McClelland et al. 2013; Reher 2020; Poulios 2010). From the perspective of valuation theory, values are not stable substances but situational claims that must be justified and negotiated – often in tension with one another in concrete disputes ( Boltanski & Thévenot 2006). Heritage values, once treated as fixed or universal, now appear shaped by fluid social constructs and shifting community relationships. As Fredheim and Khalaf (2016) and others have noted, conventional typologies often fail to reflect the dynamic hierarchies and meanings attached to heritage over time and across social groups, which is why this paper aims to shift attention from value lists to modes of value transformation.

This dynamic becomes especially evident where heritage is entangled with social transition and contestation. For example, former industrial sites, once deemed obsolete, can be revalued as carriers of community narratives, social symbolism, and labour histories ( Robson 2023). Similarly, post-conflict or post-colonial heritage illustrates how significance is repeatedly redefined or resisted by affected communities, particularly when identity and memory intersect with geopolitical transformation ( Salazar 2012). In these settings, value attribution is continuously negotiated, emerging from lived experience, trauma, reappropriation, or ideological realignment ( Smith 2006; Macdonald 2013).

These examples point to a broader need: the values assessment requires a framework that can account for multiplicity, transformation, and affective meaning, rather than translating significance into static lists. This becomes particularly pressing where heritage intersects with experiences of displacement, fragmentation, or cultural reassembly, and where affective attachments and social meanings shift faster than formal designations can follow ( Pastor et al. 2021). What follows from this is the need for iterative, pluralistic frameworks that reject static, universal interpretations in favour of situated, evolving understandings of significance ( Harrison 2013; Avrami et al. 2019).

### Rupture as a factor reconfiguring significance in architectural heritage

The twentieth century brought repeated geopolitical and social ruptures – border changes, population displacements, and mass migrations – often traumatically imposed on multiple coexisting communities. These ruptures in the continuity of historical space and time, particularly within specific territorial infrastructures, have profoundly reconfigured territorial infrastructures such as settlement patterns, property relations, and commemorative landscapes, thereby reshaping how communities relate to place and to the built environment ( Landzelius 2003; Masalha 2012, Saifi & Yüceer 2013). Across Europe and its neighbouring regions in the aftermath of World War II, such forced re-orderings altered who could inhabit, claim, and narrate architectural remnants, making the attribution of heritage significance especially unstable and contested.

**Figure 1.**
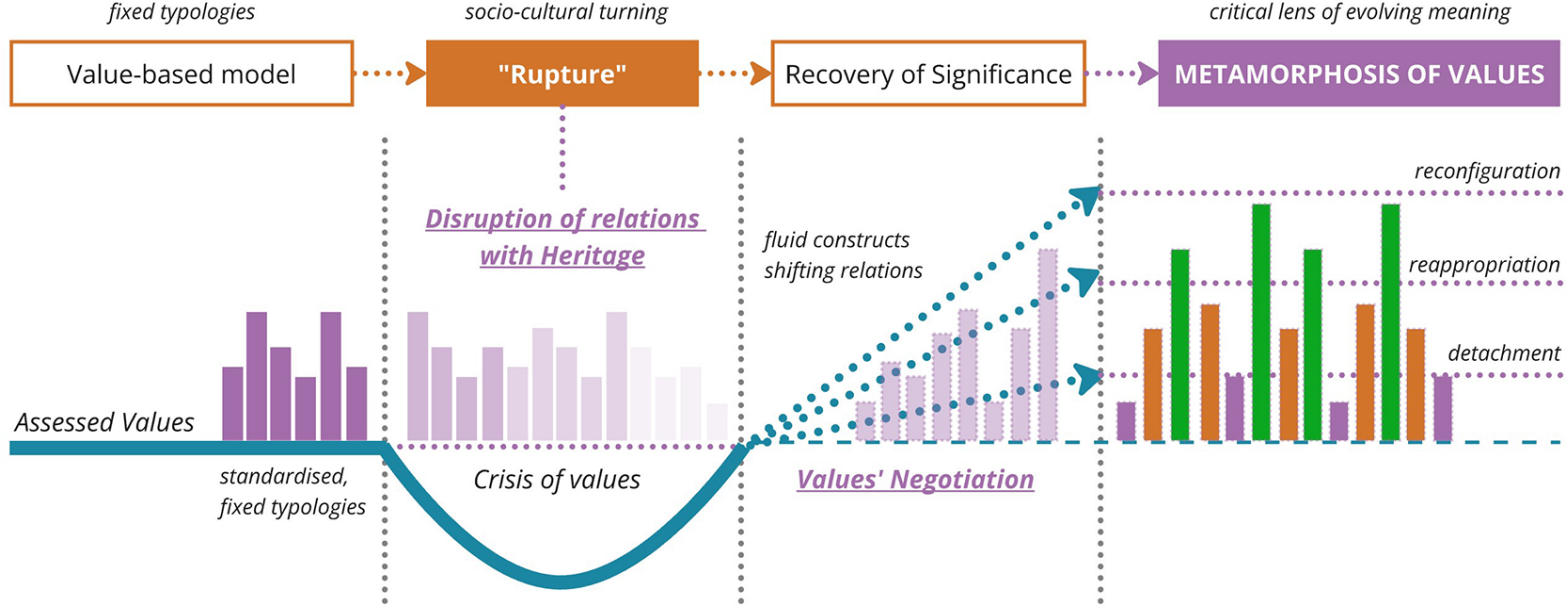
Conceptual framework supporting the metamorphic approach. (Copyright © 2025 Authors).

Such ruptures disrupt both established systems of significance and the everyday relationship between architectural heritage and the dwelling practices of affected communities. Often transmitted through memory and testimony, they persist alongside unresolved architectural remnants ( Pye 2001; Keene 2005). In these contexts, political and civic calls to remove what is perceived as “intrusive” heritage may be legitimised, yet risk erasing regional memory and rendering contemporary narratives incomplete. At the same time, such disruptions can generate transformation in significance through processes of reconfiguration, reappropriation, or detachment from their original cultural anchors, as communities renegotiate their relationship with the past. Rather than marking a definitive break, these moments often act as catalysts for reinterpretation, in which values are neither simply lost nor replaced, but transformed in response to new social and historical realities ( Figure 1).

The critique of fixed typologies is particularly relevant for vernacular, neglected, or living heritage, whose significance is often produced through ongoing use, social relations, and contested everyday practices, and therefore often fails to fit into established categories. In such cases, static frameworks may flatten or obscure the relational richness through which heritage becomes meaningful in the first place ( Olukoya 2021). Moreover, the traditional conservation goal of preserving a “comfortable past” can sideline or alienate communities seeking to redefine, repair or repurpose inherited places and meanings in the present ( Poulios 2010). This is one reason to complement value typologies with an analytic focus on how meanings accumulate, layer, and shift across time and perspectives.

Architectural heritage, especially, is a category where material permanence meets socio-cultural transformation. Buildings embody historical craftsmanship and design, yet they also evolve with their users, reflecting shifting traditions, functions, and civic meanings ( Bond & Worthing 2016). Their significance emerges through multiple registers: visual impressions ( Ahmad 2006), spatial narratives ( Walter 2013), and geo-cultural contexts that shape identity and public relevance. Taken together, these dimensions make architectural valuation particularly prone to plurality, conflict, and reinterpretation over time.

Consequently, architectural heritage resists interpretation through a single evaluative lens. Communities often adapt buildings for new uses, transforming material and symbolic functions to reflect aesthetic trends, political agendas, or emotional responses. These influences are diverse (historical, social, religious, environmental, or political) and they affect conservation priorities, especially in contexts shaped by conflict, migration, or climate change ( de la Torre 2002; Liang et al. 2023).

In such settings, values assessment helps experts select from typologies those best suited to architectural examples ( Armitage & Irons 2013). However, meaningful valorisation cannot rest on expert selection alone; it also has to resonate with communities who act as custodians, users, and interpreters of heritage meaning. Value assignment is a collective, socially negotiated process ( Throsby 2006), influenced by competing actors, including ideological successors and disengaged observers. This tension between typological selection and negotiated meaning helps explain why fixed frameworks often underperform in contested and transitional contexts.

### Heritage values: the heuristic discourse versus objectification

Yet much academic discourse still tends to privilege typology-based frameworks rooted in treaties and institutional models. As the act of naming values circulates into institutional routines, it can shift from a heuristic device into an objectified checklist – an instance of the broader process by which analytical categories become reified through institutionalisation ( Berger & Luckmann 1966), thus contributing to the ossification of heritage values ( Olukoya 2021). Standardised typologies often fail to register the contested and perspective-dependent character of significance, especially in regions shaped by displacement, community fragmentation, or cultural layering.

This limitation becomes especially acute in the assessment of *residual* and *clustered* heritage. These terms will be discussed in more detail in the subsequent parts of the paper; they describe conditions where significance emerges through partial, overlapping, or contested relationships between community and place ( Stec et al. 2025; De Marco 2025). Fixed frameworks frequently fail in such contexts, calling for more adaptive models that can accommodate narrative multiplicity and socio-cultural complexity.

The problem addressed here is therefore not that typologies are inherently flawed but – once translated into administrative routines – they are often handled as if they were stable, exhaustive, and directly operational. The critique in this paper targets this institutional hardening of value language, not the existence of values-based approaches as such. This hardening is rarely neutral; it is shaped by authority, procedural constraints, and uneven capacities to make claims legible in assessment settings. In response, this paper proposes a metamorphic lens for heritage valuation that shifts attention from enumerating “what values a site has” to describing how meanings change across time, stakeholders and contexts. The framework distinguishes three modes of transformation – *accretive* (meanings accumulate), *palimpsestic* (layers persist and may remain latent), and *refracted* (meanings diverge across perspectives) – intended as analytic prompts for interpretation. We explicitly recognise the paradox that any new vocabulary can itself become reified in practice; for that reason, the three modes are presented as heuristic instruments of reflection, meant to complicate assessment and keep contestation visible, especially in residual and clustered contexts. To make the paper’s trajectory explicit, we first outline how analytical value typologies have been translated into normative-operational templates; subsequently, we present the contexts of clustered and residual heritage, and finally, we introduce the metamorphic lens for their observation of value transformation.

## Research questions

As a conceptual contribution on values typologies and the evolving, contested significance of architectural heritage ^1^, the paper is structured around two guiding questions.

• (RQ1): Considering the fluidity of meaning attributed to architectural heritage, how can value typologies be conceptualised in terms of “evolution” or “metamorphosis”, rather than as fixed typologies?
• (RQ2): How might rethinking and updating value typologies function as an instrument of reflection in residual and clustered heritage contexts and support more inclusive, adaptive, and meaningful conservation strategies?

To address these questions, the paper pursues the following conceptual objectives:

• (O1): Critically synthesise existing frameworks for defining and applying typological values in architectural heritage, and trace how descriptive typologies are translated into normative-operational templates, drawing on interdisciplinary theories and references.
• (O2): Clarify how the paper uses the notions of residual and clustered heritage as analytic contexts, and discuss how disruption, coexistence, and plurality conditions shape the contextual limits of typology-based value assessment.
• (O3): Propose a revised conceptual model for value change in complex and layered settings by proposing the metamorphic lens as a heuristic instrument of reflection.

## Materials and methods

The methodological process comprised the following key linked steps:

1) Interdisciplinary Narrative Literature Review and Genealogical Mapping. A cross-disciplinary narrative analysis of values assessment theories was conducted to identify foundational references, prevalent value typologies, and their relative frequency and correspondence across heritage sectors.
2) Typology-Attribute Mapping. Definitions of listed value typologies were analysed and compared to extract conceptual attributes for each, forming a Value (V) > Attribute (A) matrix.
3) Contextual Application. A critical discourse was developed around residual and clustered heritage, incorporating regional contextualisation. The focus was on the dynamics experienced by these sites, the evolving perceptions of associated communities, and their relationship with heritage and valorisation processes.
4) Evaluation of a ‘Metamorphosis’ Framework. A conceptual framework was proposed for metamorphic value typologies, reflecting the fluid mechanisms influencing architectural heritage within residualisation and clustering dynamics.

### (1) Interdisciplinary narrative literature review and genealogical mapping

A cross-disciplinary analysis of values assessment theories was conducted to identify foundational references, prevalent value typologies, and their relative frequency (reported descriptively, not statistically). This step provides a foundational reference base for the subsequent typology-attribute mapping and the later conceptual synthesis.

A total of 55 sources were reviewed, including international journal articles, monographs, book chapters, and institutional reports. Selection criteria prioritised relevance to architecture and built heritage, explicitly excluding sources primarily centred on museum or intangible heritage. Importantly, the search extended beyond indexed and bibliometrically retrievable publications, incorporating older or non-indexed materials, many published before 2000 or produced by heritage institutions and associations. This corpus was assembled to ensure a comprehensive conceptual coverage and genealogical reconstruction rather than for bibliometric representativeness, which would otherwise fail to include core references at the basis of value typologies and assessment theories.

The review reconstructed a historical timeline spanning over 150 years (1849–2013) of heritage value theory development ( De Marco & Parowicz 2025a). It begins with seminal contributions from John Ruskin (1849, 1851), which represent early efforts to articulate architectural meaning through value. The period between 1980 and 2010 marks a particularly fertile era of scholarly output in values assessment, especially concerning architectural heritage. However, more recent academic production has become increasingly sector-specific, focusing on individual architectural typologies or regional cases, often at the expense of broader theoretical integration. This narrowing of scope has also been accompanied by a critical re-evaluation of values assessment itself, especially regarding its applicability in diverse socio-cultural and regional contexts.

The selected references were intentionally interdisciplinary, ensuring balanced representation across the most relevant fields: architecture, sociology, economics, and archaeology. The following proportions describe the composition of the reviewed corpus (not the distribution of the wider field) ^2^:

• The architecture discipline contributed 22 out of 55 sources (40%), reflecting its direct relevance to the cultural and functional aspects of built heritage.
• Each of the remaining fields, sociology, economics, and archaeology, contributed 11 sources (20% each), offering perspectives on civic interaction, resource valuation, and the cultural-historical depth of heritage.

**Figure 2.**
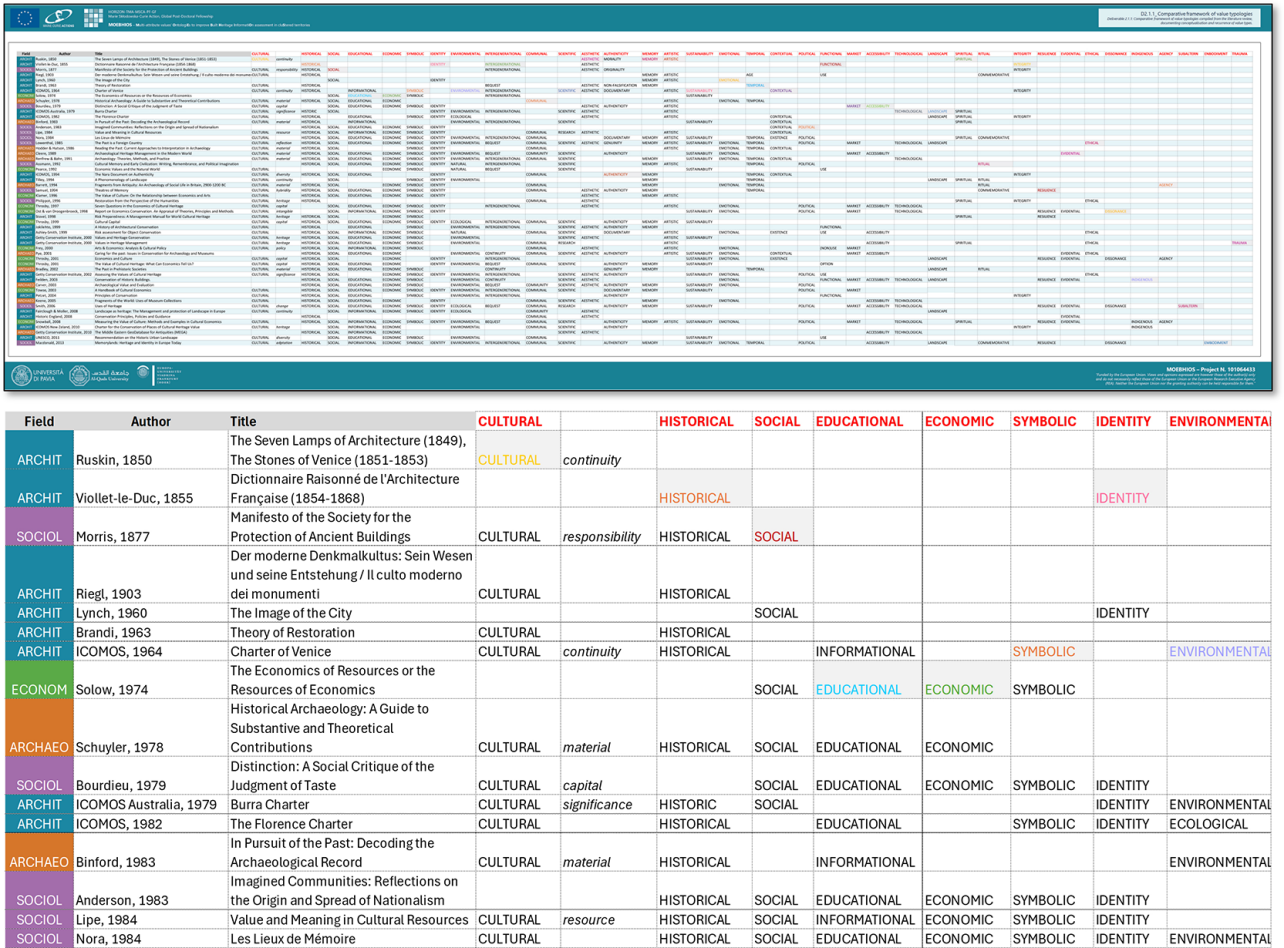
Comparative framework of value typologies compiled from the literature review. (Copyright © 2025 Authors).

The literature review concluded with the extraction of conceptualised value typologies from each source. To visualise how value typologies recur, overlap, and stabilise across disciplines and time, a comparative framework was developed. These were compiled into a comparative framework ( Figure 2), documenting the recurrence (as a descriptive mapping) of each value type and facilitating a broader analysis of the operability of the values assessment approach within architectural heritage. The resulting synthesis provided critical insights into:

• Temporal trends in value definition across different decades;
• Distribution of relevance across specific value types and disciplinary domains;
• Emerging clusters and overlaps between typologies over time.

This framework forms the analytic and conceptual foundation for the ensuing proposal of a more adaptive and dynamic system of value typologies tailored to residual and clustered architectural heritage.

### (2) Development of the Value (V) > Attribute (A) matrix

The literature review enabled the extraction and comparison of value definitions across the investigated theoretical sources, identifying key value typologies. An initial observation revealed that many of these definitions overlap across multiple categories, often referencing one another within their conceptual structures, which makes the boundaries between typologies analytically porous. For example, an architectural site may be assigned *historical* value due to its expression of *memory*; similarly, cultural assets are often attributed *symbolic* value when they convey a sense of *identity* or *communal* dimension. Likewise, *aesthetic* value is frequently associated with *artistic* qualities and is considered to embody *cultural* or *spiritual* significance. Meanwhile, *memory* is sometimes defined through references to *historical*, *cultural*, *ritual*, or *communal* elements. This observing pattern motivated the subsequent step of isolating more specific properties and qualities associated with each typology.

Building on this observation, we treated the porous boundaries between typologies as a methodological difficulty: when value categories borrow from one another, comparing frameworks at the level of labels alone risks conflating distinct logics of significance. To address this, we shifted the unit of comparison from value names to the *attributes* through which values are articulated and justified in the literature. The goal was to avoid redundancy and enhance clarity and comparability when applying value typologies to architectural heritage cases. The selection process was intentionally designed to avoid circular definitions (i.e., descriptions of a value that rely on terms conventionally associated with other values). Instead, distinctive descriptors were extracted and organised into six analytic categories, which together help construct a distinct conceptual profile for each value dimension.

• Descriptive: Attributes tied to observable traits that classify or define heritage recognisability (e.g., *distinction*, *tie*, *landmark*, *rarity*).
• Contextual: Terms reflecting how heritage is shaped by, or situated within, broader socio-cultural or spatial frameworks (e.g., *foundation, inheritance, dominance, construct*).
• Functional: Words that describe the roles heritage plays in society (e.g., *benefit*, *harmony*, *reversibility*, *inclusion*).
• Temporal: Attributes related to durability, transformation, or historical continuity (e.g., *long-term *, *investment*, *accumulation*, *sequences*).
• Perceptual: Concepts that express how heritage is experienced and internalised by individuals or communities (e.g., *essence*, *balance*, *grounding*, *resonance*).
• Instrumental: Terms linked to the capacity of heritage to inspire or enable action and change (e.g., *transmission*, *benefit*, *interconnection*, *adaptation*).

**Figure 3.**
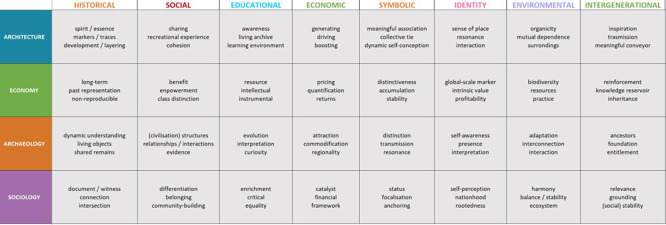
Value > Attributes matrix, organised for value typology and disciplinary category. (Copyright © 2025 Authors).

For each value typology, 12 attributes were identified (three per disciplinary category) to provide a multi-sectoral lens for analysing meaning. This step allowed for a nuanced and systematic exploration of value conceptualisation, forming the basis for a typological critique. To move beyond value labels and make their internal logic comparable, the analysis translates value definitions into an attribute-based structure. The result of this process is summarised in a Value (V) > Attribute (A) matrix ( De Marco & Parowicz 2025b), presented in Figure 3, which serves as a cornerstone for the analytical discussion that follows.

### (3) Contextual discourse on ‘Residual’ and ‘Clustered’ heritage

The discussion on the fluidity of value assessment and the evolving nature of value typologies is particularly relevant in architectural heritage contexts where significant shifts in stakeholder communities have occurred in an immovable heritage dimension. These shifts alter not only the agents responsible for valorisation but also the interpretive frames through which value is assigned, understood, and transmitted. In what follows, we therefore move from typology comparison to the socio-historical conditions under which valuation becomes especially unstable and contested.

Community movement and the resulting cultural exchange are long-standing features of heritage dynamics, especially within the Mediterranean basin. Over the past century, and particularly in the aftermath of the Second World War, population transfers and resettlements have shaped the socio-cultural history of many European and neighbouring regions. In these cases, regional community structures play a fundamental role in shaping the inhabitation and preservation of the built environment. At the same time, shifts in community composition redefine the social groups interacting with architectural heritage, which may be owned, reclaimed, or estranged from the communities’ current sense of identity.

As such, the value assigned to heritage is not static but varies, reflecting the lived experiences of communities facing voluntary or forced migration, and the interpretive frames through which architectural traces are read, used, and contested are reconfigured. These transitions affect not only the type of values attributed to heritage but also whether existing typologies remain suitable for describing the complex, often layered meanings that architectural sites accumulate over time.

Against this background, the notions of ‘residual’ and ‘clustered’ heritage are introduced as contextual lenses for analysing how co-existing communities reassign and reinterpret heritage values, often in conflicting or contested ways.

*Residual heritage*

Residual heritage refers to immovable cultural assets that remain in place but are no longer situated within the cultural, political, or legal frameworks of those who originally created or valued them. The term derives from the Latin *residuum*, meaning “that which is left behind”, capturing both the physical persistence of these sites and their detachment from former custodians. Such heritage includes structures like churches, cemeteries, civic buildings, and urban ensembles that have endured major geopolitical shifts—such as border realignments, decolonisation, or regime change—which displaced the communities or powers that once shaped their significance ( Stec et al. 2025).

**Figure 4.**
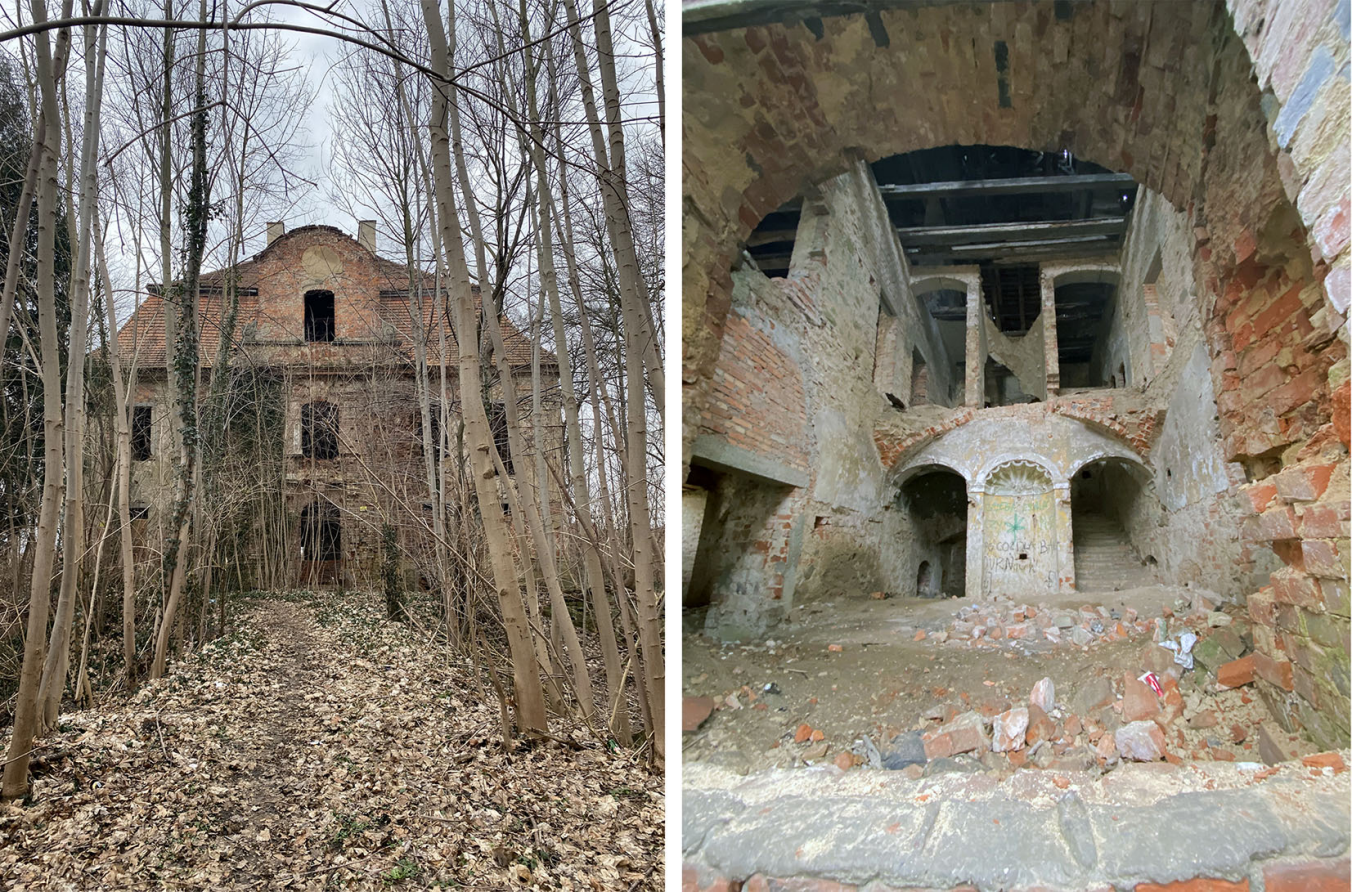
Residual heritage example: the Trzebiel palace (formerly German territory, now western Poland). (Copyright © 2025 Authors).

In such contexts, heritage remains physically in place, while the original creators or custodians (whether states, communities, or institutions) have been displaced or disconnected by geopolitical change. These former agents of significance are now geographically and politically removed, lacking authority or access. Meanwhile, current host communities have inherited the proximity to these sites without the accompanying cultural or historical frameworks. This disconnect often leads to a redefinition of meaning, where earlier values are neglected, reinterpreted, or selectively integrated into new narratives. Examples include the notable ecclesiastical heritage of the Protestant Saxon communities who voluntarily departed en masse from Transylvania in the 1990s; the post-German heritage of western Poland, now inhabited by communities relocated in 1945 from former eastern Polish territories; Polish religious and noble architecture now located in Belarus, Ukraine, and Lithuania; Serbian ecclesiastical heritage in Kosovo, currently under exclusive political control by Muslim Kosovar authorities; and colonial-era architecture in post-independence African and Latin American states ( Figure 4).

Residual heritage thus reflects complex layers of historical rupture and value transition. It embodies the enduring materiality of the past, yet its meaning is often renegotiated in the absence of uninterrupted cultural continuity. These sites challenge static or universal value typologies by highlighting how significance may fragment, linger, or be selectively recovered across communities divided by time, geography, or identity. Recognising this condition is essential for heritage assessments that aim to remain responsive to diverse historical experiences and evolving relationships between people and place.

*Clustered heritage*

Clustered heritage describes conditions arising from forced densification and the aggregation of diverse, often dissonant communities within defined territorial boundaries. These contexts are marked by extreme dynamism in the valorisation of heritage (especially architectural heritage) due to intense social, political, and cultural pressures ( De Marco 2025).

Clustered heritage reflects territorial compression, where sites become spaces of enforced coexistence. Here, architectural heritage is shaped by overlapping and often contradictory value systems, contributed by different social and cultural perspectives. The conflictual dialogue between parallel narratives often results in competing claims, contested meanings, and shifting conservation agendas, driven more by governance strategies than by community consensus. Processes such as gentrification further complicate these settings, as adaptive reuse risks erasing intangible cultural elements or displacing communities.

**Figure 5.**
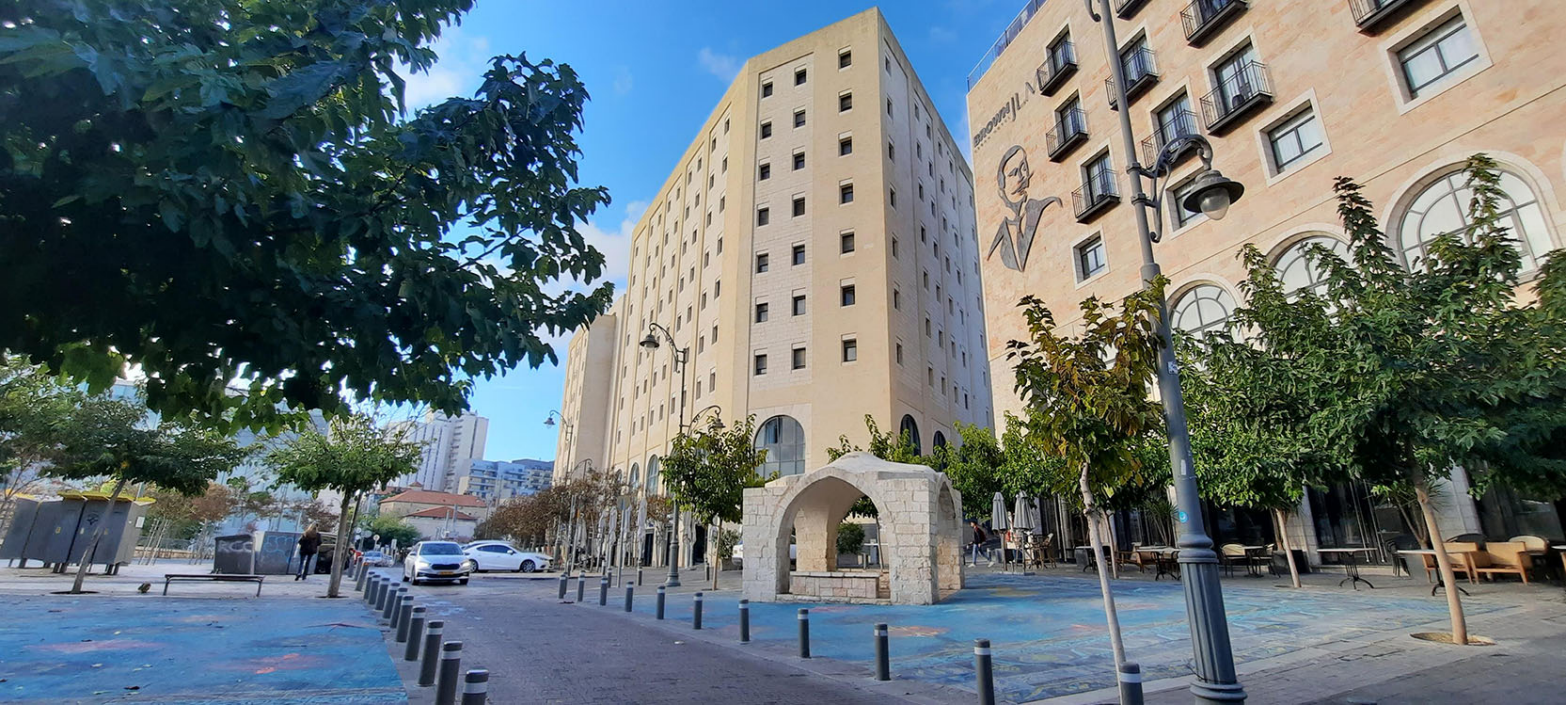
Clustering of historic architectural heritage in Jerusalem, Jaffa Street area. (Copyright © 2025 Authors).

Clustered heritage is thus understood through the lenses of spatial compression, community displacement, and overlapping cultural narratives. Architectural sites function not only as material anchors but also as symbolic constructs in the negotiation of identity and memory. The case of Palestinian architectural heritage illustrates this model, as demographic pressures and evolving geopolitical landscapes continually reshape both the interpretation and preservation of the built environment ( Figure 5).

Comparable dynamics of heritage re-signification emerge in regions where architectural landscapes have been shaped by the long-term coexistence of multiple communities under shifting political borders. In the Balkans, the Banat region exemplifies a shared architectural territory—comprising religious buildings, civic structures, rural settlements, and industrial heritage—fragmented after the First World War among Serbia, Hungary, and Romania in 1920. In Cyprus, overlapping urban fabrics of churches, mosques, and shared public spaces reflect centuries of Christian–Muslim coexistence, continuing to embody layered identities despite the geopolitical division since 1974. In Crimea, re-signification is closely linked to Crimean Tatar palatial, religious, and educational architecture, repeatedly reinterpreted as authority shifted from imperial and Soviet periods to the post-2014 context. By contrast, in the Donbass region, Soviet-era industrial, civic, and commemorative architecture has become the primary focus of contestation and reassessment amid ongoing conflict and territorial realignment. In these contexts, architectural heritage persists as a material record of plural community histories, continuously renegotiated in response to political change, displacement, and evolving forms of coexistence. Thus, valorisation is not merely about inheriting significance but about actively negotiating it. Sites that once represented a singular memory now serve as arenas for competing narratives. This directly challenges the validity of fixed, universal value typologies, which often fail to reflect the relational and processual aspects of value formation ( Poulios 2010).

Rather than rejecting values-based frameworks altogether, clustered heritage advocates for their expansion, not as the identification of a site's inherent values, but as a process of understanding how values are enacted, contested, or silenced through social interaction and visibility. This approach supports a more democratic, iterative model of value assessment, grounded in the lived realities, exclusions, and aspirations of all communities embedded within a heritage cluster.

*Shared challenges: Disconnection and variability*

Both residual and clustered heritage are marked by a distinct form of disconnection, a rupture in the continuity of custodianship and interpretation through which local heritage is ordinarily recognised and valued. This is often triggered by geopolitical decisions or compounded by social, territorial, or environmental factors, and acts as a key driver of regional transformation. These changes deeply affect cultural continuity, social resilience, and long-term sustainability.

As different communities contribute diverse and often contradictory value frameworks, their coexistence and interaction pose significant challenges for heritage interpretation. Moreover, the internalisation of heritage meaning may vary not only among groups but also across different historical moments (before and after rupture), resulting in altered perceptions and redefined relationships to heritage.

In such scenarios, value becomes inherently fluid, not only over time, but through coexisting interpretations and reconfigurations. This renders the application of static typologies problematic. Instead, the proposal to allow for variability and metamorphosis in value recognition creates room for more inclusive, community-sensitive understandings of significance, ones that honour both cohesion and cultural legacy.

### (4) Evaluation of a metamorphosis approach on values typologies

The term *metamorphic* originates from ancient Greek, combining *meta-* (μετά), meaning “after” or “change,” and *-morphic* (μορφή), meaning “form” or “shape.” It was initially used to describe physical transformation, a concept central to Ovid’s *Metamorphoses* (2–8 AD [ 1986]), which recounts around 250 myths centered on change. Over time, the term’s meaning evolved to include metaphorical and conceptual shifts. In the 19 ^th^ century, Lyell (1833) applied it in geology to describe rocks altered by heat and pressure, reinforcing its foundational association with transformation. Today, *metamorphic* also signifies profound cultural and social shifts in identity, perception, and meaning, often triggered by external influences.

Heritage values, described by Fouseki and Bobrova (2018) as *cultural meanings*, are neither intrinsic nor static. Instead, they are continuously reshaped through processes of reassessment, reinterpretation, and adaptation. The notion of *metamorphic heritage values* recognizes that significance is not a fixed property of a site, object, or tradition but a dynamic construct responsive to changing social, cultural, and political contexts. Just as metamorphic rocks are transformed under pressure, heritage values can be similarly reconfigured by external forces.

As Wells and Lixinski (2016, 352) argue, “ *heritage values are not fixed, and are best understood as processes that are in constant flux.*” Likewise, Fouseki (2022) contends that heritage values cannot be rigidly classified into discrete, self-contained categories but must be seen as interdependent and evolving, shaped by shifting contexts and continuous redefinition.

Rather than being a neutral inheritance, heritage is expected to function as an *active site of meaning-making.* Harrison (2013, 228-229) states that heritage is “ *not a passive process, but an active assembling of a series of objects, places and practices that we choose to hold up as a mirror to the present, associated with a particular set of values that we wish to take with us into the future.*” This implies that what is valued, how it is valued, and why it is valued are all subject to transformation, core to the concept of metamorphic heritage values.

The valuation process itself is metamorphic. Different stakeholders continuously assign, emphasise, or contest meanings over time. Smith (2006, 38) highlights that heritage is “ *inherently political and discordant*”, shaped by power structures, ideological shifts, and competing narratives. This resonates with Riegl’s (1903) early classification of monument values, which demonstrates how heritage is valued differently for its age, historical relevance, or current utility, depending on societal priorities. Graham et al. (2000) reinforce this by noting that heritage values are never singular or stable but always open to negotiation, reinterpretation, and contestation.

The metamorphic nature of heritage values can be highlighted in how specific attributes are emphasised, de-emphasised, or redefined according to contemporary perspectives. A site or tradition may be celebrated for its historical importance in one era, its aesthetic merit in another, and its social relevance in yet another. UNESCO (2003, Art. 2 (1)) affirms this fluidity in its definition of intangible cultural heritage, emphasising that it “ *is constantly recreated by communities and groups in response to their environment, their interaction with nature, and their history*”, thereby highlighting the continual reshaping of heritage values.

This interplay of continuity and transformation in heritage values can be further understood through three related concepts: *accretive*, *palimpsestic*, and *refracted* heritage values.

• Accretive heritage values refer to the gradual accumulation of meanings over time, where new layers are added without replacing previous ones. This process of *accretion* reflects incremental growth, integrating new elements into existing interpretive frameworks. As heritage values evolve, earlier meanings may become less prominent, subsumed, overshadowed, or relegated to the background in collective perception. Nevertheless, these prior values do not disappear entirely; they may remain latent, reemerging in specific cultural, academic, or commemorative contexts ( Ceccarelli 2017).
• Palimpsestic heritage values describe the partial overwriting of older interpretations by newer ones. The term *palimpsest* derives from manuscript practices in which earlier texts were erased and replaced, though often faintly visible beneath the new writing. In heritage, this metaphor illustrates how older meanings are not erased but are reinterpreted or obscured through contemporary lenses ( Silverman 2023).
• Refracted heritage values underscore the subjectivity of interpretation. Borrowed from optics, *refraction* refers to how light bends when it passes through different media, suggesting that heritage, too, is perceived differently depending on the observer’s standpoint. Cultural, ideological, or historical positioning influences how meaning is constructed. A single heritage element may thus carry contrasting meanings for different groups, leading to competing or parallel narratives ( Silberman 2012; Su 2018; Kandil 2024).

These three conceptual models illustrate that heritage values are rarely linear or singular. Instead, they reflect evolving frameworks of engagement, shaped by context, contested by experience, and transformed through time. The metamorphosis lens thus invites a reassessment of static typologies, supporting more responsive and pluralistic frameworks capable of accommodating heritage’s inherent fluidity.

The metamorphic approach articulates the understanding that heritage values are not static attributes but evolving constructs shaped by ongoing interaction, negotiation, and reinterpretation. As part of this research’s methodology, it offers a means to critically engage with the limitations of fixed typologies, especially in settings marked by historical rupture or contested significance. By enabling a more adaptive reading of value attribution, the approach facilitates a deeper alignment between heritage assessment and the lived realities of diverse communities, particularly in contexts where cultural meanings are layered, discontinuous, or reemerging. It thus provides a necessary conceptual foundation for reassessing value frameworks and guiding more inclusive conservation strategies.

## Results

The analysis of value typologies and the identification of metamorphic processes yielded three key findings on the transformative dynamics of value-based heritage assessment. Specifically, the results include:

1) a critical evaluation of existing value typologies;
2) the conceptualisation of metamorphic phenomena within specific categories;
3) the classification and reinterpretation of contemporary value typologies, illustrating how these can be transposed through a metamorphic lens to support a more fluid and inclusive understanding of architectural heritage.

### (1) Critical observations from the literature review

The literature review on values assessment in architectural heritage revealed important insights into both the characteristics of heritage values and the interdisciplinary nature of their conceptualisation. These observations are instrumental in guiding future debates and revisions of heritage value frameworks, particularly in contexts that challenge conventional typologies.

*Chronological evolution and disciplinary diffusion of value typologies*

**Figure 6.**
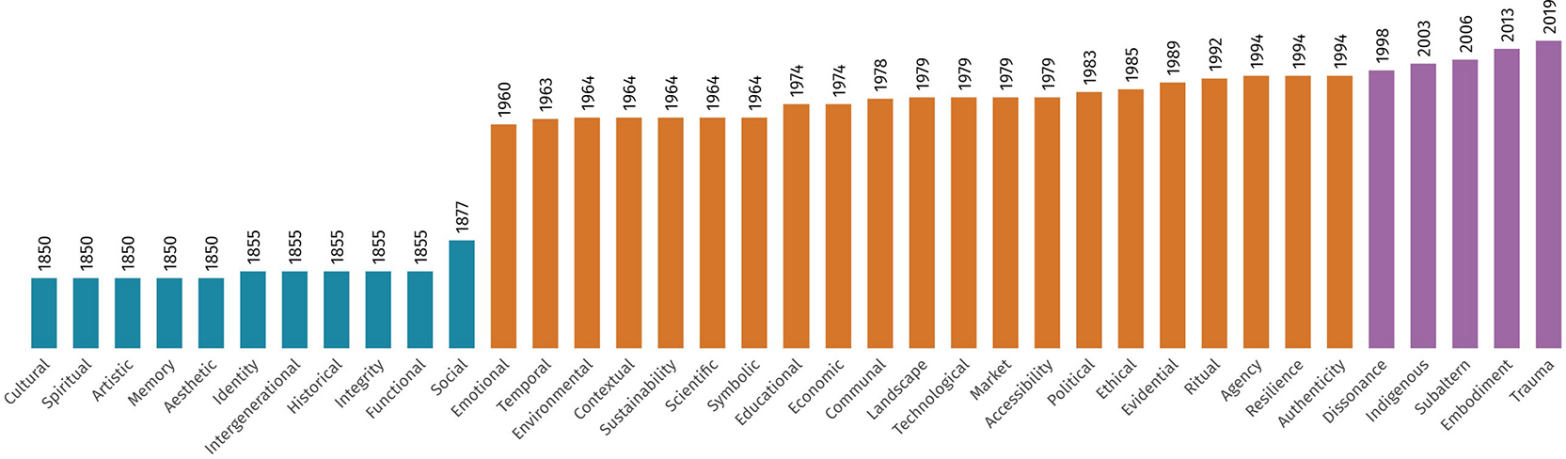
Conceptualisation of values by chronological order and phases. (Copyright © 2025 Authors).

The review revealed a chronological progression in the development of value typologies (see Figure 6), which can be grouped into three main phases:

• 1849–1900: This early phase corresponds with the institutionalisation of heritage significance, introducing foundational values such as *cultural, historical, social, identity, intergenerational, aesthetic, memory, artistic, functional, spiritual,* and *integrity.*
• 1950–1994: Reflecting the post-war reconstruction era and the democratisation of heritage, this period saw the emergence of values such as *educational, economic, symbolic, environmental, communal, scientific, authenticity, sustainability, emotional, temporal, contextual, political, market, accessibility, technological, landscape, ritual, resilience, evidential, ethical,* and *agency.*
• 1998–2019: In line with globalisation and multicultural discourses, the most recent values identified reflect a focus on perception, identity, and affect. These include *dissonance*, *indigenous*, *subaltern*, *embodiment*, and *trauma*, all of which are particularly relevant to discussions on residual and clustered heritage.

This temporal trajectory corresponds broadly to three societal shifts: nation-building and nationalism, post-war recovery and social development, and globalisation and multicultural recognition. The growing complexity and the increasing emotional and identity-based focus in more recent value categories reflect the emergence of fluidity as a critical agent in contemporary valorisation practices, reinforcing the idea of reconsidering fixed typologies.

**Figure 7.**
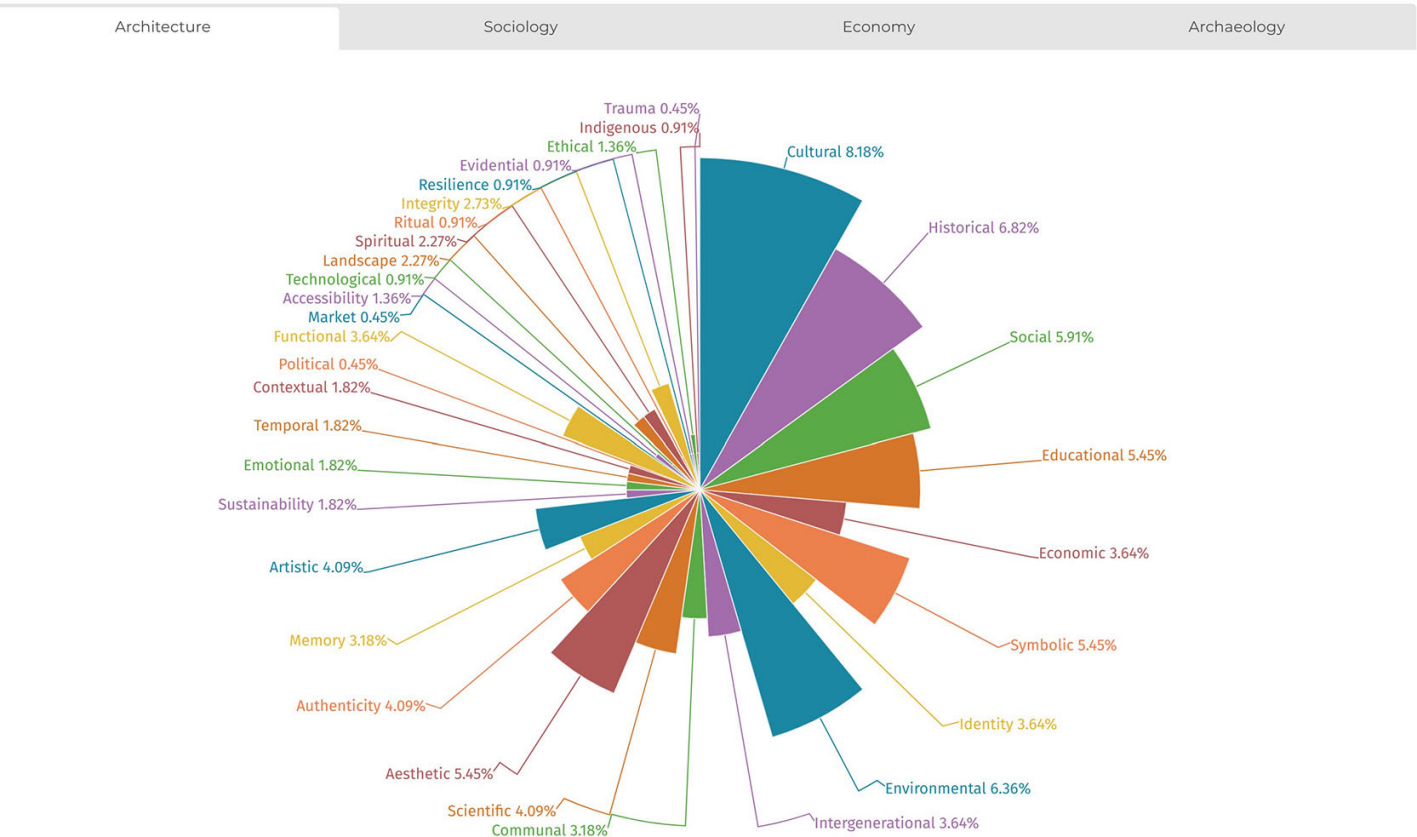
Distribution of values from references’ analysis in the field of architecture. (Copyright © 2025 Authors).

**Figure 8.**
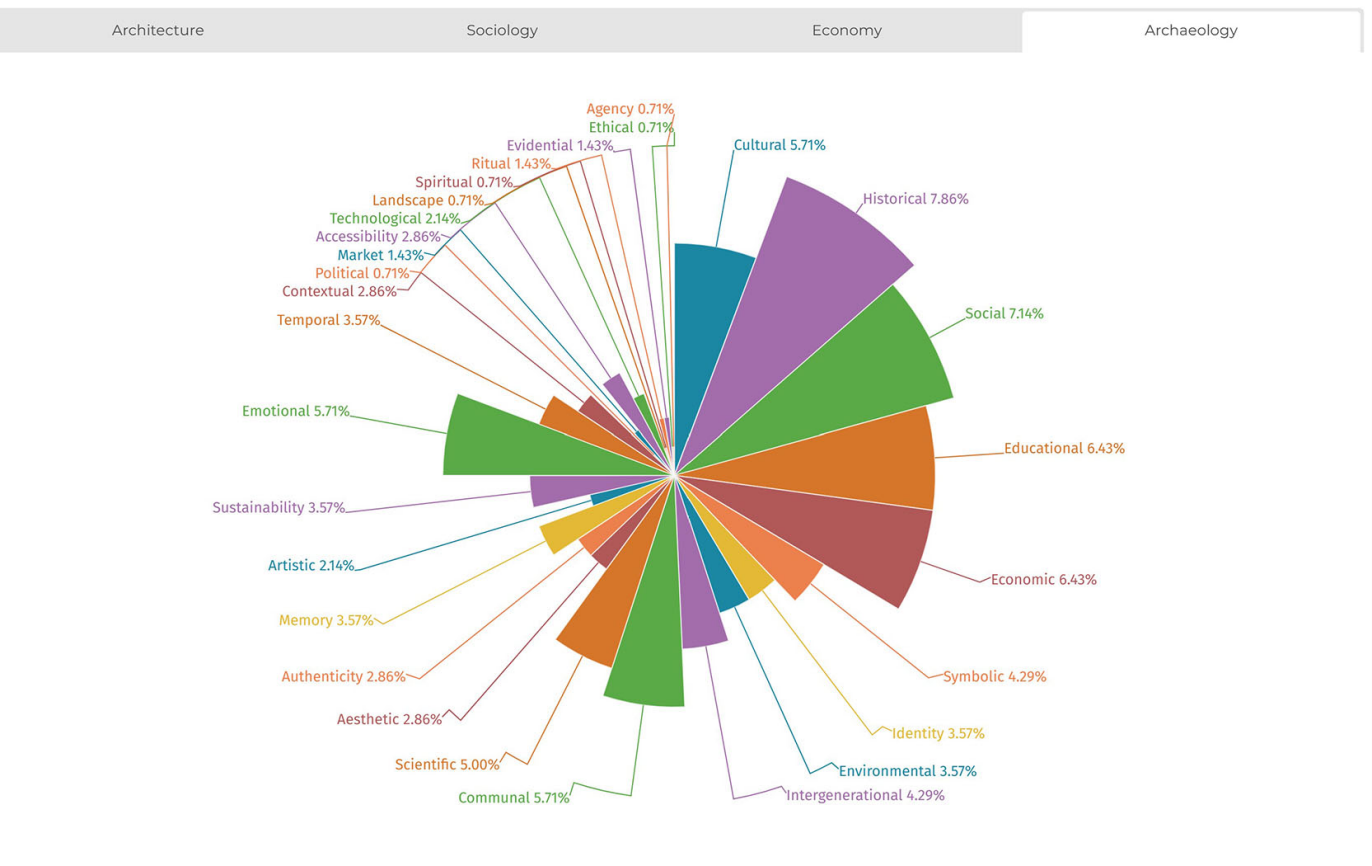
Distribution of values from references’ analysis in the field of archaeology. (Copyright © 2025 Authors).

**Figure 9.**
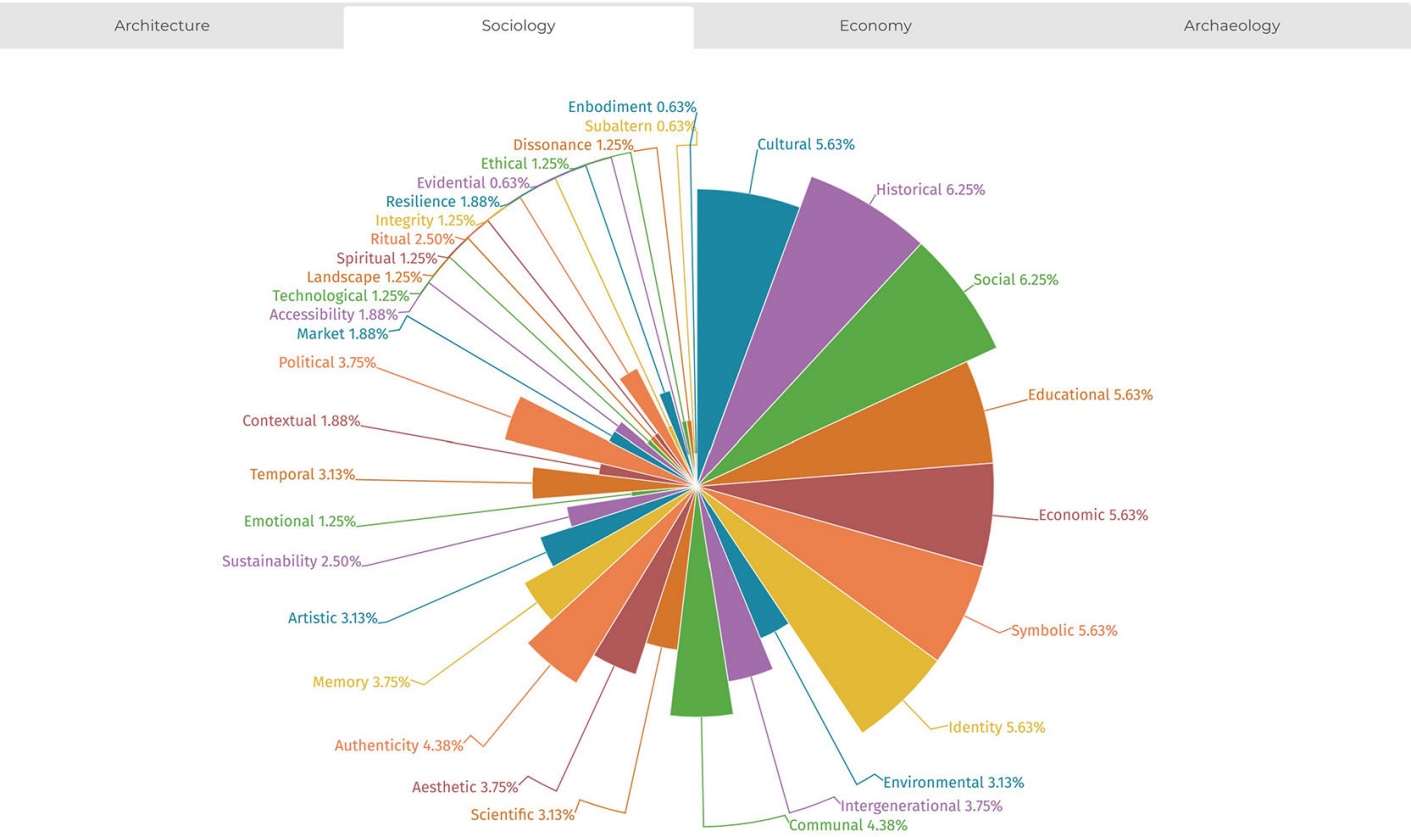
Distribution of values from references’ analysis in the field of sociology. (Copyright © 2025 Authors).

**Figure 10.**
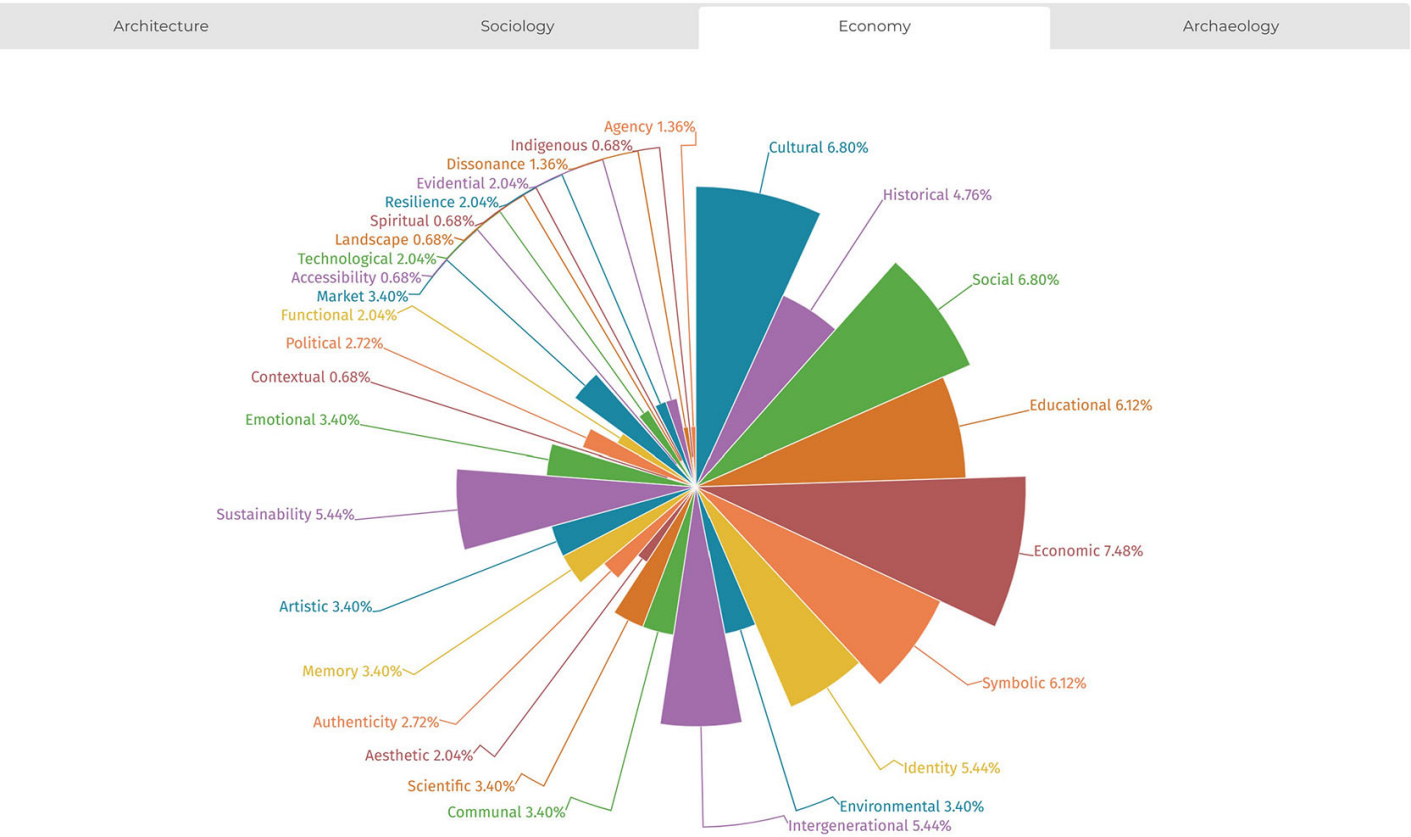
Distribution of values from references’ analysis in the field of economics. (Copyright © 2025 Authors).

The analysis also allowed for documenting the distribution of value typologies across the four disciplines examined (sociology, architecture, economics, and archaeology), highlighting that each field exhibits stronger associations with specific value types. This trend is illustrated in Figures 7- 10. The temporal distribution of typologies is also considered to help explain their varied prevalence across the historical timeline covered by the literature review.

In the humanistic fields of sociology, economics, and archaeology, certain values appear as foundational to meaning attribution. These include *cultural, historical, social, educational, economic, symbolic*, and *identity.* More recent typologies reveal stronger discipline-specific tendencies. For example, in sociology, the emphasis on *communal* values, both in the present and in terms of generational continuity, is particularly pronounced. Here, heritage valorisation is linked to the representation of political, social, and ritual dimensions, with a focus on preserving resilient and authentic memory.

In archaeology, a dual technical-emotional perspective emerges. The discipline underscores the importance of *access*, *temporal reference*, and *contextualisation* as key mechanisms through which communities relate to heritage and understand their human bonds through scientific inquiry.

In economics, the concept of value is framed in terms of *resource*, reflecting a strong link to *cultural*, *intergenerational*, and *sustainability* considerations. Economic valuation thus emphasises both heritage’s utilitarian function and its role in long-term social benefit.

Architecture, meanwhile, bridges *human* and *material* dimensions of heritage. It foregrounds *functionality* in connection with *artistic*, *aesthetic*, and *integrity*-based values. This includes concerns for *authenticity* and integrates attention to *spiritual* and *landscape* values within a broader systemic understanding of heritage.

*Quantitative diffusion of values around heritage*

**Figure 11.**
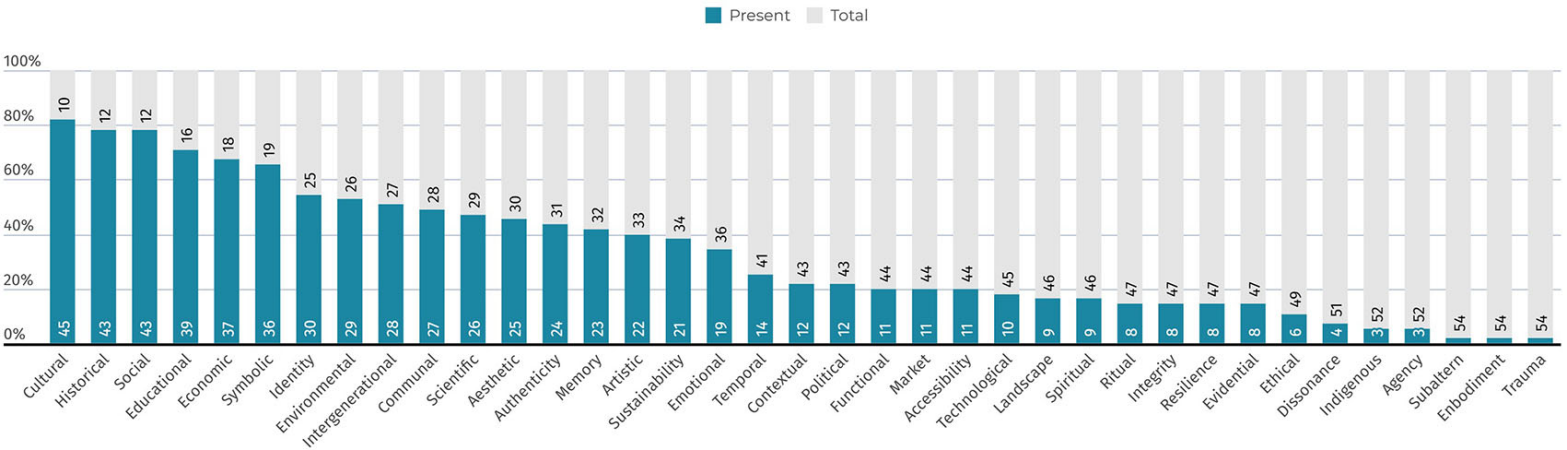
Diffusion of specific value typologies from the literature review. (Copyright © 2025 Authors).

**Figure 12.**
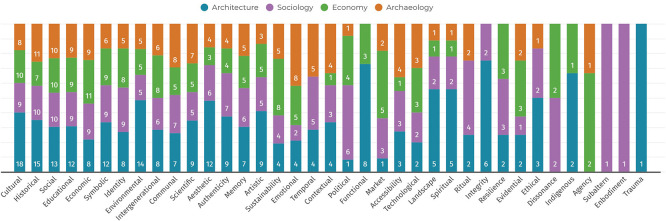
Interdisciplinary distribution of values for each typology. (Copyright © 2025 Authors).

An analysis of the recognised value typologies identified in the literature reveals both their diffusion ( Figure 11) and distribution across disciplinary fields ( Figure 12). Each value typology is visualised quantitatively based on the number of literature review sources in which it is cited. This distribution enables a clearer understanding of the disciplinary emphasis placed on specific value types.

Three main categories of diffusion emerge:

1) Balanced Distribution: These values are cited relatively evenly across all four disciplines, with each field contributing approximately 25% of the citations (in the reviewed corpus). Their broad acceptance suggests a shared conceptual foundation across domains. Examples include *cultural, historical, social, communal, artistic, scientific*, and *intergenerational.*
2) Majority Allocation in One or Two Disciplines: These values are cited primarily within one or two disciplines, accounting for over 50% of total references, and may be absent from others. This reflects a more specialised or field-specific application. Examples include *market*, *political*, *spiritual*, and *contextual.*
3) Narrow Allocation: Some values are cited only sporadically in the foundational reviewed literature. This reflects a more limited or highly contextualised conceptualisation, and in some cases, a near-isolated emergence within a single disciplinary discourse. Examples include *ethical*, *functional*, *temporal*, *dissonance*, and *subaltern* values, which have gained prominence mainly in recent critical and postcolonial heritage scholarship.

This categorisation highlights how certain values maintain interdisciplinary relevance, while others remain closely tied to specific academic or professional perspectives. Understanding this distribution is essential for identifying both convergence and fragmentation in the conceptual use of heritage values.

*Conceptualisation and variants of 'Cultural' value*

**Figure 13.**
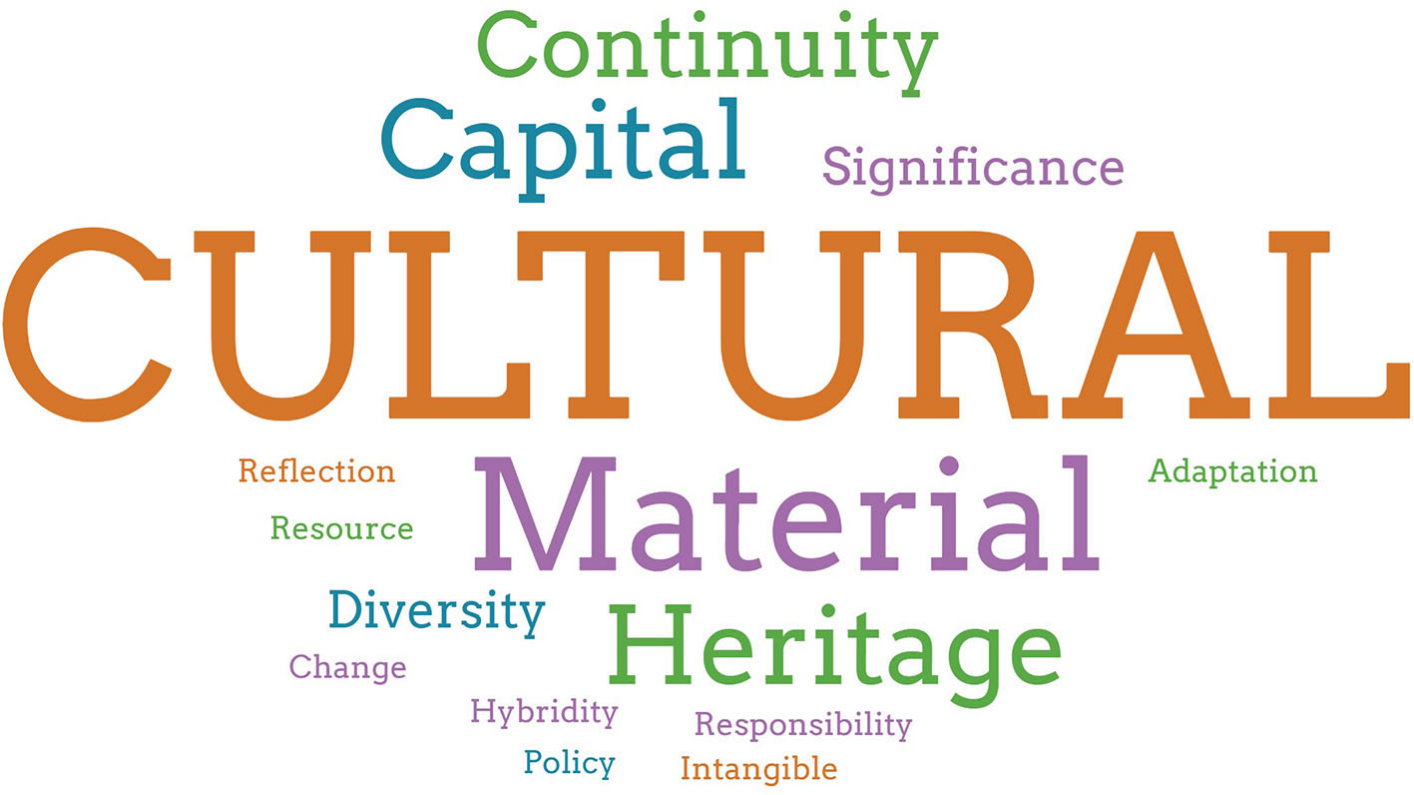
Terminological variations and compound descriptors of ‘cultural’ value. (Copyright © 2025 Authors).

The analysis of the conceptualisation of *cultural* value reveals that its usage is often both general and qualified. In many cases, the term appears on its own, while in others it is accompanied by a specification, such as *cultural continuity (* ICOMOS 1964 *)*, *cultural capital (* Throsby 2001 *)*, or *cultural diversity (* ICOMOS 1994 *).* The distribution of these variations is visualised by frequency, with the most commonly cited terms displayed in larger font sizes to indicate their relative prominence ( Figure 13).

This diversity in terminology underscores the character of *cultural* value as a compound concept **,** one that inherently encompasses multiple dimensions and interpretations. Rather than functioning as a singular or self-contained category, *cultural* value tends to incorporate complex and multifaceted attributes that reflect varied disciplinary and contextual emphases.

The findings suggest that *cultural* value, when used in isolation, may not fully capture the holistic significance attributed to architectural heritage in the reviewed corpus. Its meaning often depends on association with other conceptual domains, reinforcing the need for a multidimensional and pluralistic approach to values assessment. In this context, the compound articulation of *cultural* value enables the inclusion of diverse, evolving, and coexisting narratives.

*Conceptual similarity and aggregated designations of value typologies*

The value typologies collected through the literature review often revealed conceptual similarities between terms that appeared distinct in name. Detailed analysis of definitions allowed for the grouping of various designations under unified typologies. A key example is the value of *authenticity*, formally institutionalised by ICOMOS in the *Nara Document on Authenticity* (1994). Prior to this, the concept was referenced through terms such as *originality,* in terms of materials and provenance ( Morris 1877), *documentary,* as factual preservation ( Ashley-Smith 1999), *non-falsification* and *genuinity*, as in the distinction between original works and copies ( Brandi 1963; Lowenthal 1985; Bradley 2002). Another example is *environmental* value, encompassing *ecological* and *natural* associations, emphasising heritage’s interaction with natural systems, sustainability, and *ecological* management.

Other examples include:

• *Educational* aligns with *informational* ( Lipe 1984; Ashley-Smith 1999).
• *Intergenerational* with *bequest* and *continuity* ( Lowenthal 1985; Feilden 2003).
• *Scientific* with *research* ( Lipe 1984; Smith 2006).
• *Contextual* with *existence* ( Ashley-Smith 1999; Throsby 2001).
• *Functional* with *use* ( Riegl 1903; de la Torre 2002).
• *Ritual* with *commemorative* ( Samuel 1994; Macdonald 2013).
• *Temporal* with *age* ( Riegl 1903).

This aggregation process helped streamline the classification of heritage values, minimising redundancy and clarifying overlaps in conceptual meaning. It also enhanced the attribution analysis across disciplines and typologies, contributing to the evidence base supporting the metamorphic approach, thus reinforcing the methodological rationale for shifting from value labels to attributes in the V > A matrix.

### (2) Conceptualising the metamorphosis of values

The interpretive framework of *accretive*, *palimpsestic*, and *refracted* values reinforces the metamorphic character of heritage valuation. In contrast to fixed or essentialist models, this approach recognises that value attribution is an evolving cultural negotiation shaped by historical rupture, demographic change, political transition, and emotional reorientation. These transformative processes are particularly evident in *residual* and *clustered* heritage settings, as contexts where communities are disconnected from heritage either through displacement (residual) or forced coexistence (clustered), and where value systems evolve, fragment, or collide over time. For analytical clarity, the metamorphoses of values in these environments can be broadly grouped into three categories:

• *Evolution* – continuity with transformation
• *Replacement* – rupture and substitution
• *Reinterpretation* – continuous renegotiation of meaning

*Evolution of heritage Values: Continuity with transformation*

In both residual and clustered heritage contexts, *evolution* reflects a gradual layering of meaning over time. Even when original custodians have been separated from the site (geographically, politically, or culturally), new actors may continue to engage with the heritage in ways that retain, reinterpret, or extend its earlier significance. For example, a religious site abandoned after a geopolitical rupture may later be appreciated for its architectural integrity or commemorative function. This process aligns with the concept of *accretive values*, whereby successive interpretations accumulate without displacing prior meanings. Rather than viewing heritage through a singular lens, accretive valuation acknowledges the co-presence of diverse temporal and cultural layers that shape the ongoing relevance of a site. In this vein, the *ICOMOS Nara Document on Authenticity (1994)* underscores that heritage significance should be approached with sensitivity to the cultural context and evolving meanings assigned by communities over time.

*Replacement of heritage Values: Discontinuity and substitution*

Heritage in residual or clustered contexts can also undergo *replacement*, especially following dramatic shifts in governance, demography, or ideology. In such cases, heritage meanings may be intentionally overwritten to align with the narratives of newly dominant communities or state actors. What was once commemorated for its spiritual, ethnic, or imperial affiliations may be reinterpreted, or actively redefined, to reflect current socio-political identities. This corresponds to *palimpsestic transformation*: previous values are not entirely erased but become obscured beneath dominant reinterpretations, remaining only as fragments, traces, or marginalised memories. Whether through state-driven rebranding or local reinterpretation, palimpsestic values reflect a rupture in continuity that substitutes one heritage narrative for another. As de la Torre (2013) argues, heritage value is often shaped not only by what is preserved, but by what is recontextualised or suppressed in response to new social and political imperatives.

*Reinterpretation: Continuous renegotiation of meaning*

Beyond evolution and replacement, *reinterpretation* is the most constant and flexible mode of value transformation. In both residual and clustered scenarios, heritage is continually reframed through changing cultural, emotional, and ideological lenses. The same site may elicit pride, grief, resistance, or indifference depending on the community engaging with it. This phenomenon is encapsulated in *refracted values*, where heritage operates not as a unified symbol but as a prism through which multiple (and often conflicting) meanings are projected. In this light, heritage becomes a space of affective negotiation, where value is never static but reshaped through ongoing dialogue, memory, and perception. This aligns with Bakker’s (2011 *)* notion of heritage as a “vessel of meanings” and “vessel of values”, which different individuals and communities draw from in distinct ways, continually redefining its significance according to lived experience and collective memory.

### (3) The metamorphosis of values: Attribute-based metamorphic readings of value typologies

**Figure 14.**
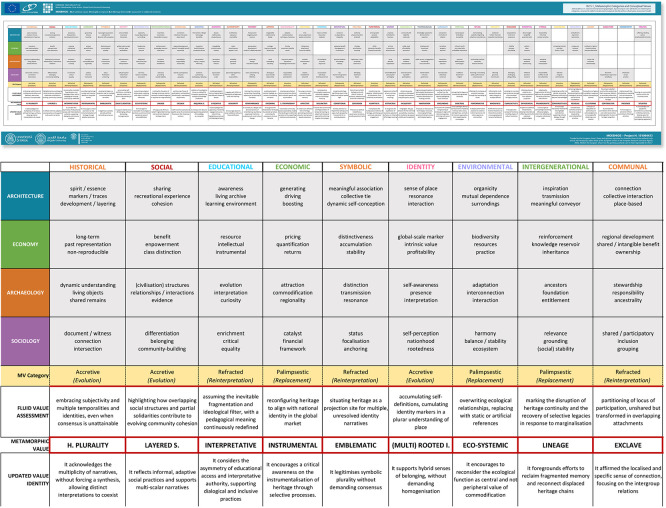
Framework of metamorphosis for value typologies. (Copyright © 2025 Authors).

This section translates the previously introduced metamorphic lens into an attribute-based interpretive tool, clarifying how existing value typologies behave under conditions of continuity, rupture, and contestation. To clarify how value typologies transform under conditions of continuity, rupture, and contestation, the following section introduces an attribute-based mapping of metamorphic modes (summarised in Figure 14).

The conceptual framework of metamorphosis, structured around *accretive*, *palimpsestic*, and *refracted* value modes, enabled a reflective re-reading of existing value typologies. Using the V>A matrix, we traced the attributes through which each typology is articulated in the reviewed literature and mapped how these attributes tend to behave in residual and clustering settings (accumulating, being overwritten, or diverging across perspectives). This step offers an interpretive mapping that clarifies how established value labels operate under conditions of continuity, rupture, and contested perception ( De Marco & Parowicz 2025c).

*Accretive mode*

For typologies that tend to operate in an *accretive* mode, reinterpretation builds upon continuity. Values retain their original foundation while integrating new layers of meaning. For example:

• *Social* may take an accretive form when overlapping social structures and partial solidarities contribute to evolving community cohesion ( *Layered Social* ). This reflects informal, adaptive social practices and supports multi-scalar narratives.
• *Sustainability* may take an accretive form when environmental care accumulates through culturally specific stewardship practices ( *Sustainable Stewardship*), linking resilience and inclusivity to local continuities.

*Palimpsestic mode*

For typologies that tend to operate in a *palimpsestic* mode, rupture leads to redefinition. Earlier meanings are not erased but become obscured, displaced, or selectively recoverable. For example:

• *Intergenerational* may take a palimpsestic form when heritage continuity is disrupted and only certain legacies are recovered or reclaimed under conditions of marginalisation ( *Lineage*). It foregrounds efforts to reclaim fragmented memory and reconnect displaced heritage chains.
• *Contextual* may take a palimpsestic form when significance is asserted despite the loss of original spatial or social logic ( *Conditional* ). It draws attention to fragmented settings and urges multi-perspective frameworks for re-contextualisation.

*Refracted mode*

For typologies that tend to operate in a *refracted* mode, value persists while interpretation varies: meanings diverge across cultural, emotional, or political perspectives. For example:

• *Symbolic* may take a refracted form when heritage becomes a projection site for multiple, unresolved identity narratives, legitimising symbolic plurality without demanding consensus ( *Emblematic*).
• *Spiritual* may take a refracted form when distinct claims of sanctity are shaped by memory, faith, access, and legitimacy, calling for inclusive approaches that respect layered cosmologies ( *Sanctual* ).

Figure 14 summarises this attribute-to-mode mapping across the analysed typologies. It should be read as a translation aid that highlights dominant metamorphic tendencies (and possible overlaps) and offers a refreshed overview of value typologies in the research context.

## Discussion

It is essential to keep the theory of heritage values up to date and responsive to the increasingly complex and dynamic realities of contemporary society. This discussion focuses on the theoretical implications of value reassessment, while also clarifying how the proposed V>A mapping and metamorphic modes can function as instruments of reflection in assessment settings. Value typologies should not remain static or universally applied but must instead evolve in dialogue with the changing socio-cultural, political, and environmental conditions that shape heritage perception and use. This requires linking theoretical developments to practical mapping actions, as locally grounded investigations of how architectural heritage is interpreted, inhabited, and negotiated in place-specific contexts.

At the core of value definition and assessment lies an inherently interdisciplinary field, shaped by the historical and theoretical trajectories of the societies that produce, preserve, and interact with cultural heritage. In the realm of architecture and the built environment, this interdisciplinarity is especially evident. The aim of preserving built heritage has always extended beyond the mere transmission of design and construction knowledge; it also encompasses the continued relevance of these structures as focal points in the evolving societal landscape – an orientation that helps explain why value vocabularies multiply and shift across disciplinary perspectives.

Architectural heritage, in particular, is among the most physically and symbolically mutable categories of cultural heritage. It undergoes continuous transformation (through adaptive reuse, structural modification, or reinterpretation of its function) to meet the evolving needs of contemporary users. These changes are also motivated by sustainability imperatives, seeking to extend the life cycle of heritage sites while minimising the consumption of resources and ensuring continuity in use.

The analysis revealed that, in the reviewed corpus, most heritage values have historically been conceptualised within the architectural domain. In contrast, values with origins in social, archaeological, and economic disciplines have emerged more recently, particularly after the 1980s. This chronological trend indicates an early focus on the physical and formal characteristics of heritage and a more recent integration of perspectives from the humanities and social sciences.

The expansion of the value discourse to include social, economic, and archaeological dimensions reflects a growing recognition that heritage is not only a physical artefact but also a social practice, an economic asset, and a bearer of cultural agency. These insights help reframe architectural heritage within broader discourses of accessibility, equity, resource use, and social resilience, thereby strengthening its relevance in the context of present-day challenges.

In this light, the application of metamorphic values (as an updated, complementary lens rather than a replacement for traditional typologies) offers a renewed, critical lens through which to assess heritage significance, supported by the V>A mapping and metamorphic framework presented earlier ( Figures 3 and 14). Particularly in residual and clustered contexts, where heritage is shaped by ruptures, overlapping narratives, and contested claims, fixed typologies often fail to capture the layered, dynamic, and context-specific nature of value. The proposed metamorphic framework aims to address this gap by acknowledging the evolving meanings attached to heritage and by offering conceptual tools for tracing how these meanings shift, resurface, or fracture over time.

Epistemologically, this approach aligns with constructivist and process-oriented understandings of heritage, where value emerges through social practice, negotiation, and temporal change rather than from intrinsic properties alone.

Moreover, adopting metamorphic values as a complementary interpretive lens contributes to future discourse as a critique of the consolidation and universalisation of value typologies in heritage policy and assessment practice. It challenges the assumption that values can be defined once and for all, or that typologies can be uniformly applied across divergent socio-political and cultural settings. Instead, it supports the development of a plural, reflexive, and adaptive model of value assessment, one that aligns with the complexities of contemporary heritage and supports more inclusive, situated, and transformative conservation strategies.

## Conclusions

This paper has deliberately positioned metamorphic values as a heuristic and methodological contribution to ongoing debates on heritage valuation, rather than as a comprehensive theoretical synthesis of all epistemological trajectories shaping the field. While the analysis acknowledges the historical consolidation of value typologies through charters and disciplinary frameworks, it does not aim to reconstruct their full intellectual genealogy, which would require a distinct and extended line of inquiry. Similarly, the metamorphic lens is not proposed as a neutral or self-sufficient solution to issues of inclusivity, but as an interpretive device intended to keep processes of transformation, negotiation, and contestation visible within values assessment. Questions of power, agency, marginalisation, and authority are therefore recognised as constitutive of heritage practice and as essential directions for further research, particularly through approaches such as critical discourse analysis and situated governance studies. The disciplinary categorisations employed in the paper serve an analytical, not reductive, function, facilitating comparison across traditions while acknowledging that contemporary heritage thinking increasingly transcends disciplinary boundaries. Future developments of this research will therefore focus on deepening the theoretical grounding of metamorphic values within critical heritage studies, and on empirically exploring how value transformation unfolds within concrete power relations, narrative struggles, and institutional settings. In this sense, the concept of metamorphic values is not presented as a closed framework, but as an open and reflexive proposition, intended to evolve alongside the complex socio-political realities in which architectural heritage is continuously re-signified.

## Ethics and consent

Ethical approval and consent were not required.

## Data Availability

Datasets on the analysis of values’ typologies have been developed as deliverables of the Horizon Europe MSCA project “MOEBHIOS” (Project No. 101064433), and are available at the Zenodo repository: Comparative Framework of Heritage Value Typologies 2025. DOI: https://doi.org/10.5281/zenodo.16755160 ( De Marco & Parowicz, 2025a) This project contains the following underlying data:
• MOEBHIOS_D2.1.1_Comparative framework of value typologies_COMPLETE.pdf To cite it: De Marco, Raffaella, and Izabella Parowicz. “Comparative Framework of Heritage Value Typologies”. Zenodo, 6 August 2025. https://doi.org/10.5281/zenodo.16755160. Data are available under the terms of the Creative Commons Attribution 4.0 International (CC-BY-4.0). Multi-Attribute Values Technical List 2025. DOI: https://doi.org/10.5281/zenodo.16756106 ( De Marco & Parowicz, 2025b) This project contains the following underlying data:
• MOEBHIOS_D2.4_MAVs Technical List.pdf To cite it: De Marco, Raffaella, and Izabella Parowicz. “Multi-Attribute Values Technical List”. Zenodo, 6 August 2025. https://doi.org/10.5281/zenodo.16756106. Data are available under the terms of the Creative Commons Attribution 4.0 International (CC-BY-4.0). Metamorphic Categories and Conceptual Values 2025. DOI: https://doi.org/10.5281/zenodo.16756803. ( De Marco & Parowicz, 2025c) This project contains the following underlying data:
• MOEBHIOS_D2.5.1_Metamorphic categories and conceptual values_COMPLETE.pdf To cite it: De Marco, Raffaella, and Izabella Parowicz. “Metamorphic Categories and Conceptual Values”. Zenodo, 7 August 2025. https://doi.org/10.5281/zenodo.16756803. Data are available under the terms of the Creative Commons Attribution 4.0 International (CC-BY-4.0).

## Footnotes

^1^
The presented research questions are not conceived as operational or empirical inquiries, but as structuring devices to organise the argument and clarify the explanatory scope of the proposed metamorphic lens. The study, therefore, adopts a methodological rather than an ontological focus: while built heritage constitutes the primary object of analysis, its significance is understood as co-produced through practices that are often categorised as intangible, including use, ritual, memory, and everyday social interaction. Rather than treating tangibility and intangibility as separate analytical domains, the paper approaches the “intangible” as an entangled register of meaning that operates through, and in relation to, architectural form. This position allows the analysis to remain grounded in architectural frameworks while accounting for the relational, processual, and socially negotiated dimensions through which heritage values are articulated and transformed.

^2^
Disciplinary allocation is used here as a provenance indicator (where a source is primarily situated), not as a claim that concepts “belong” to a single field. We recognise that theoretical influences and critical perspectives routinely cross disciplinary boundaries and are often shared across fields.
