# Supplementary material for: Metamorphic heritage values: Revisiting architectural heritage assessment in clustered and residual contexts
Source: Open Res Eur. 2026 Feb 16;6:51. [Version 1] doi: 10.12688/openreseurope.23051.1 (PMC13100613; doi:10.12688/openreseurope.23051.1)
Supplement: Peer review history

# Reviewer report: Reviewer response for version 1

**DOI:** 10.21956/openreseurope.24932.r71906

**Published:** 2026-04-23

**Authors:** Cristina Gonzalez-Longo

**Affiliations:** 1 University of Strathclyde, Glasgow, Scotland, UK

This paper offers a different approach to the assessment of heritage values, which is always welcomed, but it does not give clear evidence to support it. The context and scope are also unclear. It states to deal with “heritage valuation and conservation practice”, which, although obviously connected, there are two distinctive areas of research. This is critical as the paper includes a series of statements that needed to be substantiated, for example, that existing practices “risk shifting from analytical descriptors into normative-operational checklists.”

 The scope, evidence, research methodology and results needed to be better explained. The paper relies on published research only, rather than including also existing architectural heritage, designation and architectural conservation policies and practices, vital for the research topic. The theoretical framework needs also revision as architectural conservation is a discipline developed over more than 200 years, something that does not seem to be recognized by the authors.

 Fundamental contributions, such as that of Cesare Brandi are not considered.

 The literature review was limited to 55 sources; it is not clear how they were selected and current legislation on designation and policy papers and guidelines used by the competent national and local authorities has not been reviewed. Perhaps an undeclared finding is that research, policy and practice are quite detached. If existing practices were researched, the authors would have seen that  is not about “rest on expert selection”, as they state, but about using scientific methodologies to evaluate heritage. This cannot be replaced by communities’ views, although they have to be also integrated within these methodologies.

 More concerning for research on heritage is the focus only in our contemporary society, as the very nature of heritage is not only to serve current needs, but, crucially, its transmission to the future. Heritage is not a commodity but a cultural asset. It is also unfair to characterise current designation legislation and methodologies of evaluation as “static lists”, state that there is “the traditional conservation goal of preserving a ‘comfortable past’ “,  that “academic discourse still tends to privilege typology-based framework s rooted in treaties and institutional models” or that “ These statements cannot be included in a scientific paper without clear evidence, as they can be quite damaging for the field.

 The very quantitative approach in the methodologies, as represented in Figures 7-12 do not appear appropriate, and conflicts with the initial intention of a more flexible conceptualisation.

 Terminology is also an issue, as it is not totally clear what the authors mean by ‘value typologies’. More critical is the consideration that the pursue of a scientific method to evaluate and protect architectural heritage is an ‘institutionalisation’.

 As people in the field of architectural heritage and conservation recognises, there is no need to create new vocabulary, but to clarify existing terms and how they are used. The offered terms of ‘residual’ and ‘clustered’ heritage and the three proposed modes of transformation do not seem to be justified, or indeed, necessary. There is a current tendency to give adjectives to heritage, which create inappropriate classifications, distracting the more comprehensive understanding of the heritage.

 The conclusions are too short in comparison with the extent of the paper, and they do not summarise the main findings.

 The writing is convoluted, perhaps the result of the use of AI to check it, which gives many times over-complicated texts.

 The figures need also some improvements: first table in Figures 2 and 14 are illegible and Figures 7-12 are excessive and not easy to read.

If any, are all the source data and materials underlying the results available?

Partly

Is the work original in terms of material and argument?

Partly

Is the work clearly and cogently presented?

No

Is the argument persuasive and supported by evidence?

Partly

Does the research article contribute to the cultural, historical, social understanding of the field?

Partly

Does it sufficiently engage with relevant methodologies and secondary literature on the topic?

Partly

Reviewer Expertise:

Architectural Design for the Conservation of Built Heritage, Conservation Theory and History, Architectural History, Sustainability, Environmental Design. History of Construction, Architecture

I confirm that I have read this submission and believe that I have an appropriate level of expertise to confirm that it is of an acceptable scientific standard, however I have significant reservations, as outlined above.

# Reviewer report: Reviewer response for version 1

**DOI:** 10.21956/openreseurope.24932.r71898

**Published:** 2026-04-21

**Authors:** Iasmina Onescu

**Affiliations:** 1 Politehnica University of Timisoara, Timisoara, Romania

**Title:** Metamorphic heritage values: Revisiting architectural heritage assessment in clustered and residual contexts

 Values-based approaches are central to architectural heritage conservation, yet their institutionalisation has often transformed analytical value typologies into normative-operational templates. This stabilisation risks obscuring the dynamic, negotiated, and context-dependent nature of heritage significance, particularly in settings shaped by rupture, displacement, and coexistence. Such limitations are especially evident in residual and clustered heritage contexts, where architectural heritage persists amid discontinuity, layered memories, and competing narratives. This study addresses the need for a more flexible conceptualisation of value attribution capable of accounting for transformation, plurality, and perspective-dependence.

 The paper presents an interesting investigation into architectural heritage conservation research field, addressing a relevant problem. The authors demonstrate a strong understanding of the field and an appropriate selection of literature, relevant for the investigated topic.

 The article is written concisely and clearly, and summarizes the results of a substantial volume of information. The statements are correctly supported with results and explanations.

 I have the following recommendations for the authors:

- Please further develop the state-of-the-art part and offer a synthesis of the findings (a table or a visual representation of the main ideas), to highlight the different approach of this paper; 
- Please clarify the research gap identified after the state-of-the-art analysis;
- Please reduce the conceptual density, as many parts of the paper seems highly abstract and dense; try to translate theoretical ideas into more direct architectural language, to improve readability;
- Please strengthen the methodological transparency, as some analytical steps are just briefly described, missing clearer description;
- Please adjust the discussion topic, because at this point it seems descriptive rather than critically interpreting the findings;
- Please clearly summarise the main contributions and explain how this research results could be used in teaching, research or practice;
- Please clearly state the limitations and opportunities of the presented work.

I do recommend reconsidering the paper after minor revision, because the topic is interesting and the work of the authors is important.

If any, are all the source data and materials underlying the results available?

Yes

Is the work original in terms of material and argument?

Yes

Is the work clearly and cogently presented?

Yes

Is the argument persuasive and supported by evidence?

Partly

Does the research article contribute to the cultural, historical, social understanding of the field?

Yes

Does it sufficiently engage with relevant methodologies and secondary literature on the topic?

Yes

Reviewer Expertise:

Heritage preservation, historical architecture

I confirm that I have read this submission and believe that I have an appropriate level of expertise to confirm that it is of an acceptable scientific standard, however I have significant reservations, as outlined above.

## References

[1] : Towards a More Cohesive and Accessible City Centre: Bridging the Gap Between Historical Identity and Modern Community’s Needs—Case Study: Lugoj City, Romania. *Heritage* .2025;8(10) : 10.3390/heritage8100396 10.3390/heritage8100396

[2] : Architectural Heritage Conservation and Green Restoration with Hydroxyapatite Sustainable Eco-Materials. *Sustainability* .2025;17(13) : 10.3390/su17135788 10.3390/su17135788

[3] : The Impact of Timber Roof Framework Over Historical Masonry Structures. *IOP Conference Series: Materials Science and Engineering* .2019;603(4) : 10.1088/1757-899X/603/4/042030 10.1088/1757-899X/603/4/042030

# Reviewer report: Reviewer response for version 1

**DOI:** 10.21956/openreseurope.24932.r71905

**Published:** 2026-04-17

**Authors:** Athena Moustaka

**Affiliations:** 1 University of Salford, Salford, England, UK

The paper offers a thoughtful (and timely) contribution to ongoing debates around value-based approaches in heritage studies, particularly in relation to their institutionalisation and the risks this poses for missing nuance, contextual sensitivity, and interpretive flexibility. The question of whether values are formalised within policy and institutional frameworks may lose their fluidity and contextual grounding, is well-articulated.

 The paper is guided by two key research questions. First, how heritage values can be conceptualised as fluid rather than fixed. Second, how such values can operate as instruments of reflection within residual and clustered heritage contexts. These questions are clearly framed and respond to important gaps in current heritage discourse, particularly in relation to how values are selected, translated, and operationalised as they migrate from theory into policy and practice.

 To address these questions, the paper begins with a review of heritage approaches over time, mapping temporal trends and identifying overlaps and shifts in emphasis. This section is particularly strong. The temporal mapping effectively illustrates how heritage paradigms have evolved and how value-based approaches have gained prominence, especially within an increasingly globalised context. It also helps to pinpoint why these discussions are becoming more pressing today. Importantly, the paper moves beyond symbolic interpretations of heritage and foregrounds embodied practices, allowing cultural, historical, economic, educational, and social dimensions, as well as identity, to emerge more clearly across different contexts. This represents a valuable shift in perspective.

 The development of the value–attribute matrix represents another key contribution of the paper. By attempting to map values and their associated attributes, the framework provides a useful tool for structuring analysis and encouraging reflection. The author notes that this framework is not intended to capture everything, which is an important clarification. This point could, however, be made even more explicit within the overall argument. Existing value-based approaches can at times become reductive, particularly when they are translated into institutional or policy frameworks. For this reason, the paper could benefit from stating more clearly that the proposed framework is not intended to capture everything, but rather to open up a broader and more reflective way of engaging with heritage values.

 The section defining residual and clustered heritage is well-developed and supported by clear examples. These concepts are used effectively to demonstrate how heritage operates in layered, contested, and evolving ways. The discussion of creative, palimpsestic, and refracted heritage further strengthens the argument, supporting the idea of heritage as inherently dynamic and subject to ongoing transformation. The categorisation of metamorphoses into evolution, replacement, and reinterpretation is particularly helpful in structuring this discussion and offers a clear analytical lens for understanding change over time.

 At the same time, the emphasis on rupture, transformation, and political contestation in space raises an important question that could be addressed more explicitly. To what extent might such frameworks inadvertently legitimise the removal or marginalisation of elements considered “obtrusive”? While the paper successfully argues for a more fluid and adaptive understanding of heritage, engaging more directly with this potential implication would strengthen its critical depth, particularly in relation to conservation ethics and decision-making processes.

 In terms of presentation, the paper is well structured, clearly written, and easy to follow. The flow of arguments is coherent, and the progression from literature review to conceptual framework and applied examples is logical and effective. The temporal mapping of heritage trajectories is a particularly strong element and stands out as a key contribution.

 One minor point concerns the inclusion of the word cloud on descriptors of "cultural value", which does not appear to add significant analytical value to the discussion. The arguments are already well supported through more substantive methods, and this element could either be further developed or reconsidered.

 Overall, this is a strong and engaging article that critically examines established approaches to heritage and challenges consolidated ways of thinking. It makes a valuable contribution by questioning how values are defined and operationalised, and advocating for more reflective and flexible frameworks moving forward. The paper is especially effective in highlighting the need to reconsider heritage as a dynamic and evolving construct within a globalised context. With some further clarification around the practical implications of its arguments, particularly regarding the potential consequences of transformation and removal, it has the potential to offer an even more impactful contribution to the field.

If any, are all the source data and materials underlying the results available?

Yes

Is the work original in terms of material and argument?

Yes

Is the work clearly and cogently presented?

Yes

Is the argument persuasive and supported by evidence?

Yes

Does the research article contribute to the cultural, historical, social understanding of the field?

Yes

Does it sufficiently engage with relevant methodologies and secondary literature on the topic?

Yes

Reviewer Expertise:

Architecture, heritage studies, and design research, particularly in relation to cultural heritage, spatial interpretation, and critical approaches to the built environment.

I confirm that I have read this submission and believe that I have an appropriate level of expertise to confirm that it is of an acceptable scientific standard.

# Reviewer report: Reviewer response for version 1

**DOI:** 10.21956/openreseurope.24932.r69866

**Published:** 2026-03-09

**Authors:** Vinh An Le

**Affiliations:** 1 Industrial University of Ho Chi Minh City, Ho Chi Minh City, Ho Chi Minh, Vietnam

**General Comments:**

 The article proposes the concept of metamorphic heritage values as an interpretive lens for reconsidering approaches to the assessment of architectural heritage in complex contexts, particularly in clustered and residual heritage settings. From an academic perspective, the study represents a commendable effort to critique the limitations of traditional value classification systems, which have often been employed as relatively stable frameworks in heritage conservation practice. The central argument of the paper—that heritage meanings are dynamic, context-dependent, and continuously negotiated among social actors—aligns well with contemporary trends in critical heritage studies. By shifting the analytical focus from fixed categories of value to the processes through which values transform, the paper offers a noteworthy conceptual contribution that expands the interpretive capacity for understanding heritage contexts characterized by layered memories and competing narratives.

 A major strength of the paper lies in its interdisciplinary and conceptually structured approach. Through a narrative literature review and a genealogical mapping of value frameworks across multiple academic fields, the study identifies overlaps and permeability among different systems of value classification. The development of the Value–Attribute (V>A) matrix provides a useful analytical tool for identifying shared conceptual characteristics and zones of intersection among various research traditions. On this basis, the identification of three modes of value transformation—accretive, palimpsestic, and refracted—gives the proposed theoretical framework a certain degree of illustrative clarity and interpretive capacity for explaining the transformation of heritage meanings in complex socio-political contexts.

 However, from the perspective of academic review, the paper also reveals several limitations that merit consideration. First, although the study constructs a relatively sophisticated conceptual foundation, the linkage between the proposed theoretical framework and empirical evidence remains somewhat limited. The different modes of value transformation are described largely at a conceptual level, while their illustration through concrete case studies or practical situations has not been sufficiently developed. As a result, the verifiability and practical applicability of the metamorphic values framework in heritage assessment remain largely exploratory.

 Second, although the paper acknowledges the role of power relations, agency, and marginalization in processes of heritage valuation, these aspects are mainly presented as directions for future research rather than being deeply integrated into the current analysis. In complex heritage contexts—particularly those involving contested memories or competing narratives—political and institutional factors often play a direct role in shaping value formation. Therefore, further clarification of how governance structures and power dynamics influence the “metamorphosis” of heritage values would strengthen the explanatory capacity and comprehensiveness of the proposed framework.

 Third, the concept of metamorphic values is presented as an open interpretive tool, which helps avoid the risk of creating yet another rigid classificatory system. Nevertheless, this openness also raises questions regarding its conceptual distinctiveness in relation to existing approaches in critical heritage studies, such as theories of layered memory, heritage discourse, or context-dependent reinterpretation of values. Clarifying the conceptual boundaries of metamorphic values and its relationship with these existing theoretical frameworks would help strengthen the academic autonomy and originality of the proposal.

 Overall, the paper provides a meaningful conceptual contribution to ongoing debates on the epistemology of heritage value assessment, particularly in complex heritage contexts characterized by multiple historical layers. Although it would benefit from stronger empirical grounding and deeper engagement with issues of power and institutional dynamics, the concept of metamorphic heritage values has the potential to serve as a valuable analytical lens, expanding value-based approaches in both research and practice in the conservation of architectural heritage.

**Advantages:**

- Contribution of a new concept: The paper proposes the concept of *metamorphic heritage values* as a new analytical lens, shifting the focus from categorizing values to understanding the processes through which heritage values evolve over time and across contexts.
- Clear critical approach to traditional value classification systems: The study convincingly demonstrates that institutionalized value classification systems are often static and normative, highlighting the need for a more flexible approach.
- Interdisciplinary research methodology: The paper synthesizes value frameworks from multiple fields, including architecture, archaeology, sociology, and economics, providing a broad and comparative theoretical foundation.
- Clear conceptual analysis tool: The construction of a Value–Attribute (V>A) matrix helps identify overlaps between different value classification systems and supports systematic analysis.
- Identification of three specific value transformation mechanisms: The three forms of transformation – accretive, palimpsestic, and refracted – offer a clear interpretive framework for understanding changes in heritage meaning.
- High level of academic reflexivity: The paper does not impose the new concept as a replacement classification system but presents it as an open interpretive tool, avoiding the risk of creating another rigid normative framework.
- Significance for critical heritage studies and conservation practice: This approach better explains complex heritage contexts where multiple layers of memory, discourse, and stakeholder groups coexist.

**Disadvantages:**

- Lack of empirical evidence: The paper mainly develops arguments at a conceptual and theoretical level, while concrete case studies or empirical data illustrating the metamorphic values framework are limited.
- Practical applicability not clarified: Although a new interpretive lens is proposed, the paper does not clearly specify how this conceptual framework can be concretely applied in heritage assessment or management processes.
- Power and governance aspects not deeply analyzed: Issues of power, agency, and discourse conflicts are only mentioned as directions for future research, rather than being directly integrated into the analysis.
- Conceptual boundaries not entirely clear: The concept of metamorphic values overlaps in some ways with existing approaches in critical heritage studies (e.g., layered memory or heritage discourse), but the academic distinctions are not sufficiently highlighted.
- High level of abstraction but limited specificity: The types of value transformation (accumulation, layering, refraction) are described rather generally, making verification or application to specific heritage contexts still in need of further development.

**Suggested Improvements:**

1. **Incorporate case studies:** The paper should include one or two specific heritage cases to illustrate how accumulated, layered, and refracted values manifest in practice. This would make the metamorphic values framework more convincing and empirically verifiable.
2. **Clarify practical applicability in heritage conservation:** The study could propose a specific procedure or analytical steps for applying the metamorphic values lens in heritage assessment, thereby increasing the conceptual framework’s practical relevance.
3. **Clarify conceptual boundaries with existing approaches:** The paper should more clearly compare the concept of metamorphic values with related theories in critical heritage studies, highlighting the novelty and unique contribution of this theoretical framework.
4. **Integrate power and governance aspects more deeply:** Analyzing how power relations, institutional structures, and discourse conflicts affect value transformation would help the framework better reflect the realities of heritage practice.
5. **Develop a visual analytical model:** The paper could include diagrams or conceptual models showing the process of transformation and interaction among different types of values, helping readers visualize and apply the framework.

These improvements would enhance the study’s empirical grounding, conceptual clarity, and practical applicability, thereby further highlighting the paper’s scientific contribution.

If any, are all the source data and materials underlying the results available?

Yes

Is the work original in terms of material and argument?

Yes

Is the work clearly and cogently presented?

Partly

Is the argument persuasive and supported by evidence?

Partly

Does the research article contribute to the cultural, historical, social understanding of the field?

Yes

Does it sufficiently engage with relevant methodologies and secondary literature on the topic?

Yes

Reviewer Expertise:

Architectural Heritage;  Historic Urban Cities; Cultural Heritage Conservation and Tourims Development.

I confirm that I have read this submission and believe that I have an appropriate level of expertise to confirm that it is of an acceptable scientific standard, however I have significant reservations, as outlined above.
